# Pregnancy-associated alterations in circulating vitamin D metabolites: higher total 25-hydroxyvitamin D and 1,25-dihydroxyvitamin D without increased free 25-hydroxyvitamin D

**DOI:** 10.3389/fendo.2026.1957418

**Published:** 2026-10-22

**Authors:** Hui-fang Zhang, Xin Chen, Jia-yi Xiang, Ze-yu Zhang, Yvonne Liu, Katrin Huesker, Volker von Baehr, Saban Elitok, Bernhard Karl Krämer, Christoph Reichetzeder, Berthold Hocher

**Affiliations:** 1 Fifth Department of Medicine (Nephrology/Endocrinology/Rheumatology/Pneumology), University Medical Centre Mannheim, University of Heidelberg, Mannheim, Germany; 2 Institute for Clinical Research and Systems Medicine, Health and Medical University, Potsdam, Germany; 3 Department of Nephrology and Endocrinology, Klinikum Ernst von Bergmann, Potsdam, Germany; 4 Institute of Medical Diagnostics (IMD), Berlin, Germany; 5 Reproductive and Genetic Hospital of CITIC-Xiangya, Changsha, China; 6 Institute of Reproductive and Stem Cell Engineering, National Health Commission (NHC) Key Laboratory of Human Stem Cell and Reproductive Engineering, School of Basic Medical Science, Central South University, Changsha, China

**Keywords:** 1,25-dihydroxyvitamin D, 25-hydroxyvitamin D, free 25-hydroxyvitamin D, gestation, pregnancy, vitamin D-binding protein

## Abstract

**Background:**

Interpretation of vitamin D biomarkers during pregnancy remains challenging because physiological adaptations alter total and free vitamin D metabolites differently, while evidence comparing multiple circulating vitamin D metabolites within the same clinical setting remains limited.

**Methods:**

We conducted a large real-world laboratory-based cross-sectional study using routine clinical data from women aged 18–45 years tested at a German diagnostic center between 2014 and 2020. Total 25-hydroxyvitamin D [25(OH)D], free 25-hydroxyvitamin D, and total 1,25-dihydroxyvitamin D [1,25(OH)_2_D] concentrations were compared between pregnant and nonpregnant women and across gestation using age-adjusted regression models. Complementary random-effects meta-analyses were performed for free 25-hydroxyvitamin D and total 1,25-dihydroxyvitamin D.

**Results:**

After exclusions, 101,454 nonpregnant and 16,017 pregnant women comprised the overall study population, with analyte-specific sample sizes reflecting availability in routine clinical practice. In age-adjusted analyses, pregnancy was associated with higher total 25(OH)D concentrations (adjusted difference 22.07 nmol/L; 95% CI, 21.50–22.63; P<0.001) and markedly higher total 1,25(OH)_2_D concentrations (adjusted difference 125.20 pmol/L; 95% CI, 115.39–135.02; P<0.001). Both metabolites were higher at later gestational stages in cross-sectional analyses. In contrast, directly measured free 25(OH)D did not differ significantly between pregnant and nonpregnant women (adjusted difference, -2.63 pmol/L; 95% CI, -8.99 to 3.74; P = 0.417). Complementary meta-analyses showed higher total 1,25(OH)_2_D and modestly lower free 25(OH)D concentrations during pregnancy; sensitivity analyses excluding the current cohort yielded similar overall patterns.

**Conclusions:**

Pregnancy was associated with higher total 25(OH)D and markedly higher total 1,25(OH)_2_D. No statistically significant difference in free 25(OH)D was detected in the much smaller available subgroup; this finding should be considered exploratory and requires confirmation in larger studies. These results highlight the importance of analyte-specific interpretation of vitamin D biomarkers during pregnancy.

## 1 Introduction

Vitamin D is a key regulator of calcium and phosphate homeostasis, but its biological actions extend beyond mineral metabolism through vitamin D receptor (VDR)-mediated signaling in multiple tissues. These actions include regulation of immune and inflammatory pathways, reproductive physiology, and placental function, providing a broader molecular context for the role of vitamin D in pregnancy (1–3). Pregnancy induces marked adaptations in vitamin D metabolism to support maternal calcium balance and fetal skeletal development. Circulating 1,25-dihydroxyvitamin D [1,25(OH)_2_D] rises early in gestation, often reaching concentrations two- to threefold higher than those observed in nonpregnant women, reflecting enhanced renal and extrarenal calcitriol production (4–8). Concurrently, estrogen-driven increases in vitamin D-binding protein (VDBP) expand the circulating binding capacity for vitamin D metabolites, leading to higher total concentrations of both 25-hydroxyvitamin D [25(OH)D] and 1,25(OH)_2_D without necessarily increasing the circulating free fraction (9–11).

These physiological changes complicate the interpretation of vitamin D biomarkers during pregnancy. Total 25(OH)D remains the standard clinical marker, but pregnancy-associated increases in VDBP may weaken the relationship between total and free 25(OH)D (9–11). Similarly, because total 1,25(OH)_2_D rises physiologically during pregnancy, a concentration considered normal according to a nonpregnant reference interval may be relatively low for a given gestational stage (4–8). Pregnancy-specific interpretation therefore requires consideration of the measured metabolite, the analytical method, its bound and free fractions, and gestational age. These complexities underscore the need to evaluate different vitamin D metabolites individually rather than assuming that they change in parallel during pregnancy.

Pregnancy complications may also disturb these adaptations. Hypertensive disorders of pregnancy and preeclampsia are characterized by placental dysfunction, systemic inflammation, endothelial activation, and changes in maternal renal physiology (8, 12). Gestational diabetes and maternal obesity have also been associated with altered vitamin D status, although these relationships may reflect complex interactions involving insulin resistance, chronic inflammation, adiposity, supplementation, and lifestyle factors (13–15). Renal dysfunction may impair 1α-hydroxylation, whereas liver disease, protein loss, and systemic inflammation may alter VDBP synthesis or binding (11). These relationships are likely bidirectional; therefore, associations between altered vitamin D biomarkers and adverse pregnancy outcomes should not automatically be interpreted as causal.

Vitamin D insufficiency is common among women of reproductive age, but prevalence varies by geographical region, season, ultraviolet-B exposure, skin pigmentation, diet, supplementation, body mass index, and the threshold used to define deficiency (16–18). Natural dietary sources are limited, and supplementation practices differ considerably. Increases in total 25(OH)D during pregnancy may reflect supplementation, altered VDBP concentrations, or both, and may not indicate a proportional increase in free 25(OH)D. Current guidance supports empiric vitamin D supplementation during pregnancy and suggests against routine 25(OH)D testing in generally healthy pregnant individuals without an established clinical indication (19).

Available studies comparing vitamin D metabolites between pregnant and nonpregnant women have important limitations. Many have included relatively small, selected cohorts, assessed only one gestational period, and used heterogeneous analytical methods. This is particularly relevant for free 25(OH)D, which has been measured directly in some studies but calculated from total 25(OH)D, VDBP, and albumin in others (9–11). Differences in VDBP assays, calculation equations, and assumptions regarding binding affinity may produce substantial discrepancies between measured and calculated concentrations. Studies of 1,25(OH)_2_D also vary in assay platform, gestational timing, supplementation patterns, population characteristics, and control selection (4–8). Previous meta-analyses have primarily focused on total 25(OH)D deficiency, pregnancy complications, or supplementation and have not comprehensively synthesized contemporary evidence comparing both free 25(OH)D and total 1,25(OH)_2_D between pregnant and nonpregnant women. A large real-world analysis can provide statistical power and routine-care evidence, whereas a complementary meta-analysis can assess external consistency across populations, assays, and study designs.

To address these knowledge gaps, we evaluated circulating total 25(OH)D, free 25(OH)D, and total 1,25(OH)_2_D in parallel analyte-specific subcohorts of pregnant and nonpregnant women of reproductive age using a large routine laboratory database. Where sufficient data were available, we also examined cross-sectional patterns according to gestational age. In a complementary systematic review and meta-analysis, we synthesized published evidence comparing free 25(OH)D and total 1,25(OH)_2_D between pregnant and nonpregnant women and performed sensitivity analyses excluding the current cohort to assess the robustness of the pooled estimates. We hypothesized that pregnancy would be associated with higher total 25(OH)D and markedly higher total 1,25(OH)_2_D concentrations, but not with a corresponding increase in free 25(OH)D.

## 2 Methods

### 2.1 Clinical observational study

#### 2.1.1 Study design and ethical approval

This was a retrospective, cross-sectional observational study based on real-world clinical laboratory data. The study was conducted in accordance with the Declaration of Helsinki and approved by the Institutional Review Board of the Institute of Medical Diagnostics (IMD) Berlin-Potsdam (approval number: IMD-2023-09; approval date: 4 October 2023). The requirement for informed consent was waived because the study used fully anonymized retrospective data from routine clinical laboratory testing and related internal quality-control procedures for assay stability, with no additional sample collection or participant contact. Because the data were retrospective and cross-sectional rather than longitudinal, within-person changes over time could not be assessed.

#### 2.1.2 Data source and participant selection

Blood samples submitted to the IMD for routine clinical analysis between March 2014 and July 2020 were screened for eligibility (**Figure 1**). Samples originated from outpatient practices and medical care centers throughout Germany, as described previously (20–22). The dataset was derived from routine clinical laboratory testing and therefore does not represent a population-based sample.

**Figure 1:**
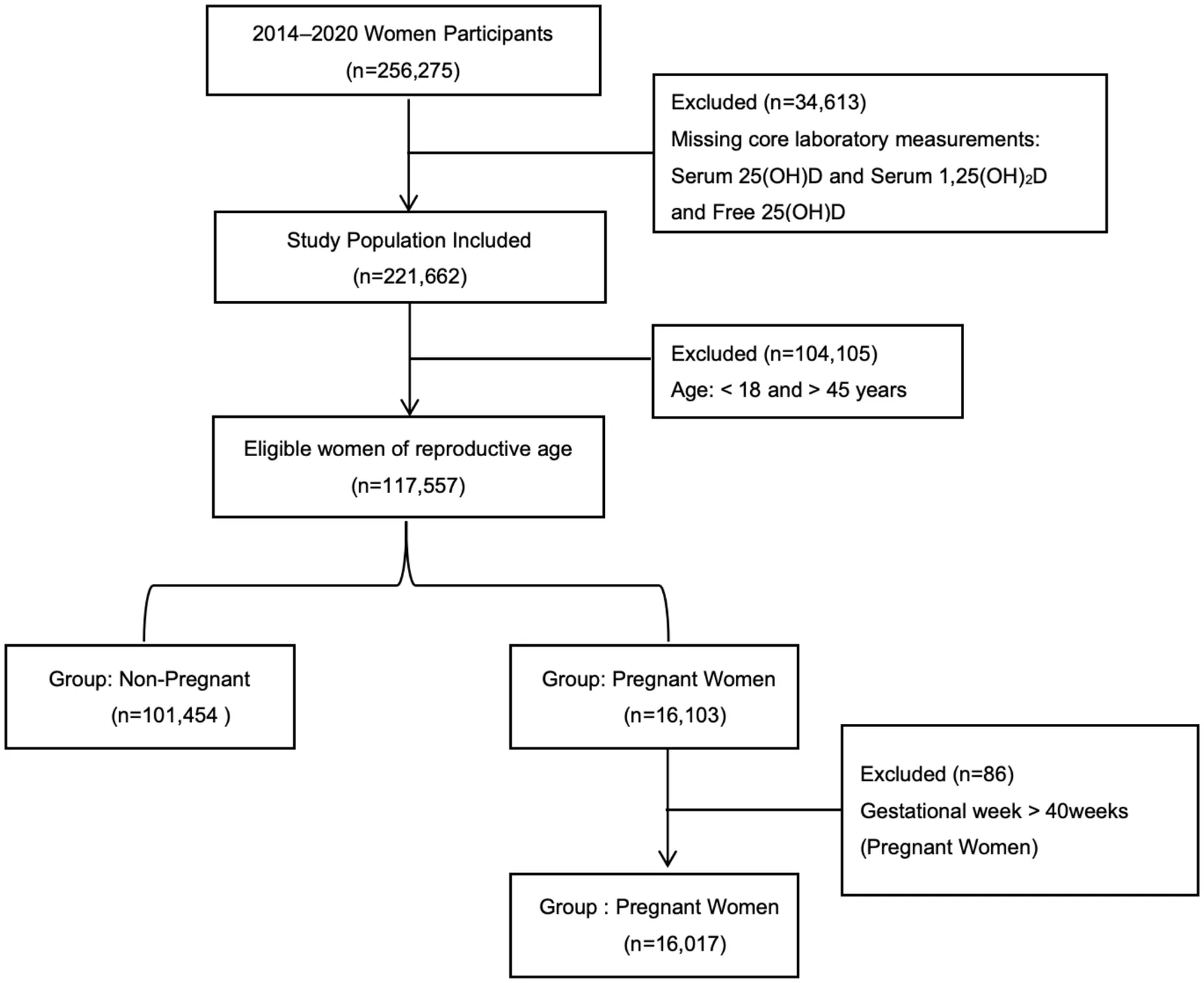
Flow diagram of study selection and analyte-specific analytic cohorts. Female individuals undergoing vitamin D testing at the Institute for Medical Diagnostics (IMD) Berlin-Potsdam between March 2014 and July 2020 were screened. After exclusion of individuals with missing core vitamin D measurements and restriction to women of reproductive age (18–45 years), participants were classified by pregnancy status. Pregnant women past the 40th gestational week were excluded.

1. Individuals without a valid measurement of any of the three vitamin D metabolites were excluded (*N* = 34,613). Because total 1,25(OH)_2_D and free 25(OH)D were ordered selectively rather than systematically in routine clinical practice, analyses of these metabolites were restricted to analyte-specific subcohorts. For each analyte, all participants meeting the overall study eligibility criteria and having a valid measurement for that analyte were included; no additional clinical selection criteria were imposed by the investigators. Clinical indications for these tests were not recorded in a sufficiently standardized manner and therefore could not be used to characterize indication-related selection.
2. Individuals aged < 18 years or > 45 years were excluded to restrict the study population to women of reproductive age, leaving 221,662 women in the initial eligible population. Participants were then classified as pregnant or nonpregnant. Pregnant participants with a recorded gestational age >40 weeks were excluded because such values were considered potentially atypical or inaccurately recorded.

Extreme values were handled at the analyte level using literature-informed physiological thresholds. Serum total 25(OH)D concentrations > 375 nmol/L were treated as missing for analyses involving total 25(OH)D (23). For total 1,25(OH)_2_D and free 25(OH)D, all observed concentrations were retained in the primary analyses irrespective of pregnancy status. Sensitivity analyses evaluating alternative analyte-specific high-value handling strategies are provided in the **Supplementary Materials**.

#### 2.1.3 Stratification and definitions

Pregnant participants were stratified by gestational age and maternal age. Gestational stages were defined according to standard obstetric criteria as first trimester (T1, ≤ 13 weeks + 6 days), second trimester (T2, 14 to 27 weeks + 6 days), and third trimester (T3, ≥ 28 weeks until delivery).

#### 2.1.4 Clinical and laboratory measurements

Total 25(OH)D concentrations were measured using the Abbott Architect i2000 electrochemiluminescence immunoassay (Abbott Laboratories, Wiesbaden, Germany), for which published analytical validation reported repeatability CVs of ≤2.3% and total imprecision of ≤6% (24). Free 25(OH)D concentrations were measured using an ELISA, as previously described (25). Total 1,25(OH)_2_D concentrations were measured using the LIAISON^®^ XL 1,25 Dihydroxyvitamin D assay (DiaSorin, Saluggia, Italy), an automated chemiluminescent immunoassay (CLIA), for which published analytical validation reported intra-assay imprecision of 1.4–5.2% and total imprecision of 3.8–7.1% (26). Laboratory-specific intra- and inter-assay CVs for the full retrospective study period could not be reconstructed; therefore, the precision estimates reported above represent published assay-performance characteristics rather than study-specific IMD quality-control data.

#### 2.1.5 Statistical analysis

Continuous variables are presented as medians (interquartile range, IQR) and categorical variables as counts and percentages. Normality was assessed using the D’Agostino-Pearson omnibus test, and all continuous variables showed significant deviation from a normal distribution (*P* < 0.05). Vitamin D metabolite concentrations were compared between pregnant and nonpregnant women using the Mann–Whitney U test. Age-adjusted linear regression models were used to evaluate associations between pregnancy status or gestational trimester and metabolite concentrations, with nonpregnant women as the reference group. Maternal age was included as the prespecified adjustment variable because it was available in a sufficiently complete and standardized manner across the routine laboratory dataset, whereas data on diet, supplementation, body mass index, ethnicity, season, comorbidities, and pregnancy complications were not available. Linear regression assumptions were assessed using residual diagnostics, including evaluation of residual distributions and homoscedasticity; the latter was additionally assessed using the Breusch–Pagan test. Potentially influential observations were evaluated using Cook’s distance, leverage, and studentized residuals. Linear regression was used to estimate adjusted mean differences, and the results were interpreted together with sensitivity analyses using log-transformed outcomes. To assess whether the imbalance in sample sizes between pregnant and nonpregnant groups influenced the estimated pregnancy-associated differences, we additionally performed repeated equal-size random subsampling. To assess the comparability of the analyte-specific subcohorts with the broader study population on available characteristics, participants with and without total 1,25(OH)_2_D or free 25(OH)D measurements were compared separately among pregnant and nonpregnant women. Cross-sectional patterns of total 25(OH)D according to gestational age were examined using nonlinear smoothing models, whereas age-adjusted linear regression was used to quantify the association between gestational week and total 1,25(OH)_2_D. The relationship between serum total 25(OH)D and total 1,25(OH)_2_D concentrations was evaluated using correlation analyses and pregnancy-status-specific linear regression models. In participants with both metabolites available, the molar total 25(OH)D/total 1,25(OH)_2_D ratio was additionally evaluated according to pregnancy status and gestational trimester. Further details are provided in the **Supplementary Materials**.

Statistical analyses were performed using R version 4.5.2 (R Foundation for Statistical Computing, Vienna, Austria) and GraphPad Prism version 10.0 (GraphPad Software, San Diego, CA, USA). *P* < 0.05 was considered statistically significant.

### 2.2 Systematic review and meta-analysis

This complementary systematic review and meta-analysis was prospectively registered in PROSPERO (CRD420261366190), conducted according to a prespecified protocol, and reported in accordance with the Preferred Reporting Items for Systematic Reviews and Meta-Analyses (PRISMA) guidelines (27). The quantitative synthesis incorporated the present cohort together with eligible previously published studies. Sensitivity analyses excluding the present cohort were performed to assess the robustness of the pooled estimates. The literature search covered studies published through February 2026.

#### 2.2.1 Search strategy, study selection, and data extraction

PubMed, Web of Science, and Embase were systematically searched for English-language studies of vitamin D metabolites during pregnancy. The search strategy combined Medical Subject Headings (MeSH) and free-text terms related to vitamin D metabolites and pregnancy. Reference lists of included studies were manually screened for additional eligible articles, and conference abstracts and prospective trial registries were also searched for potentially relevant records. Detailed search strategies and additional information on study selection, handling of multiple reports from the same cohort, and data extraction are provided in the **Supplementary Materials**.

Duplicate records were removed before screening. Remaining records were screened by title and abstract for potential relevance. Full texts were obtained for records that appeared to meet the eligibility criteria or for which eligibility could not be determined from the title and abstract alone. Full-text articles were then assessed against predefined inclusion and exclusion criteria. When multiple publications reported data from the same study population, the most comprehensive or most recent report was retained for data extraction, while related publications were reviewed for **Supplementary Information**.

Study screening, full-text eligibility assessment, and data extraction were performed independently by two investigators, with disagreements resolved by discussion and consensus. For the systematic review, extracted data included study design, population characteristics, gestational window, assay method, and key findings. For the meta-analysis, sample sizes and summary statistics required for effect-size calculation were also extracted.

#### 2.2.2 Eligibility criteria

Eligible studies were original peer-reviewed human observational studies reporting circulating free 25(OH)D and/or total 1,25(OH)_2_D concentrations during pregnancy. Included studies were original peer-reviewed human observational studies reporting free 25(OH)D and/or total 1,25(OH)_2_D in pregnancy. Animal and *in vitro* studies, review articles, conference abstracts, editorials, commentaries, case reports, and intervention studies primarily designed to investigate vitamin D supplementation were excluded.

For quantitative synthesis, studies were additionally required to include a nonpregnant comparison group or provide sufficient comparative data between pregnant and nonpregnant women. When studies reported multiple physiological states or multiple comparisons derived from longitudinal or cross-sectional data, one prespecified independent comparison between pregnant and nonpregnant women was selected for the primary meta-analysis to avoid duplicate inclusion of participants.

#### 2.2.3 Quality assessment

Methodological quality of the studies included in the meta-analysis was assessed independently by two investigators using the Joanna Briggs Institute (JBI) critical appraisal checklists appropriate to each study design. Analytical cross-sectional studies and cohort studies were assessed using the corresponding JBI checklists. Any disagreements were resolved by discussion and consensus.

#### 2.2.4 Data analysis for meta-analysis

A random-effects meta-analysis was conducted to compare circulating free 25(OH)D and total 1,25(OH)_2_D concentrations between pregnant and nonpregnant women. When studies reported multiple groups or gestational stages, one prespecified independent comparison was selected for the primary analysis. If necessary, reported summary statistics were converted to means and standard deviations using established methods. Both free 25(OH)D and total 1,25(OH)_2_D concentrations were expressed in pmol/L.

The ratio of means (ROM) was calculated on the logarithmic scale. Random-effects models were fitted using restricted maximum likelihood with Knapp-Hartung adjustment. Results were back-transformed and presented as ROM (pregnant/control) with 95% confidence intervals. Statistical heterogeneity was evaluated using the I² statistic and interpreted according to Higgins et al. (28). A two-sided *P* value < 0.05 was considered statistically significant. Reporting bias was assessed using funnel plots and Egger’s test (**Supplementary Figure S3**). To assess the influence of including the present cohort on the pooled estimates, sensitivity analyses were performed after excluding it, thereby restricting the meta-analysis to previously published independent studies. The detailed results are provided in the **Supplementary Materials**. Robustness analyses also included leave-one-out analyses and influence diagnostics (**Supplementary Figure S4**). Further details regarding unit conversion, handling of reported summary statistics, analyte-specific sensitivity analyses, and meta-analytic influence diagnostics are provided in the **Supplementary Materials**. All meta-analyses were performed using R software (version 4.5.2; R Foundation for Statistical Computing, Vienna, Austria).

## 3 Results

### 3.1 Results of the clinical observational study

After excluding individuals with no available measurement of any of the three vitamin D metabolites, 221,662 women remained eligible. Restriction to participants aged 18–45 years and exclusion of pregnant participants with a recorded gestational age >40 weeks yielded 101,454 nonpregnant and 16,017 pregnant women (**Figure 1**). Analyte availability differed because total 25(OH)D was routinely measured, whereas total 1,25(OH)_2_D and free 25(OH)D were ordered selectively. No participant had valid measurements of all three vitamin D biomarkers simultaneously; therefore, a complete-case analysis directly comparing all three metabolites within the same individuals was not feasible.

**Figures 2A–F** shows the distributions of total 25(OH)D, total 1,25(OH)_2_D, and free 25(OH)D according to pregnancy status. For total 25(OH)D, data were available for 101,104 nonpregnant women and 15,938 pregnant women; median (Q1, Q3) concentrations were 57.50 (37.50, 77.50) and 80.00 (60.00, 100.00) nmol/L, respectively (**Figures 2A, B**). For serum 1,25(OH)_2_D, data were available for 5,952 nonpregnant women and 183 pregnant women; corresponding values were 126.72 (96.24–168.00) and 259.20 (202.80, 312.00) pmol/L (**Figures 2C, D**). For free 25(OH)D, data were available for 304 nonpregnant women and 60 pregnant women; corresponding values were 18.18 (13.05, 30.00) and 19.62 (15.09, 27.75) pmol/L (**Figures 2E, F**). Both serum total 25(OH)D and total 1,25(OH)_2_D were right-skewed, whereas free 25(OH)D showed a narrower distribution.

**Figure 2:**
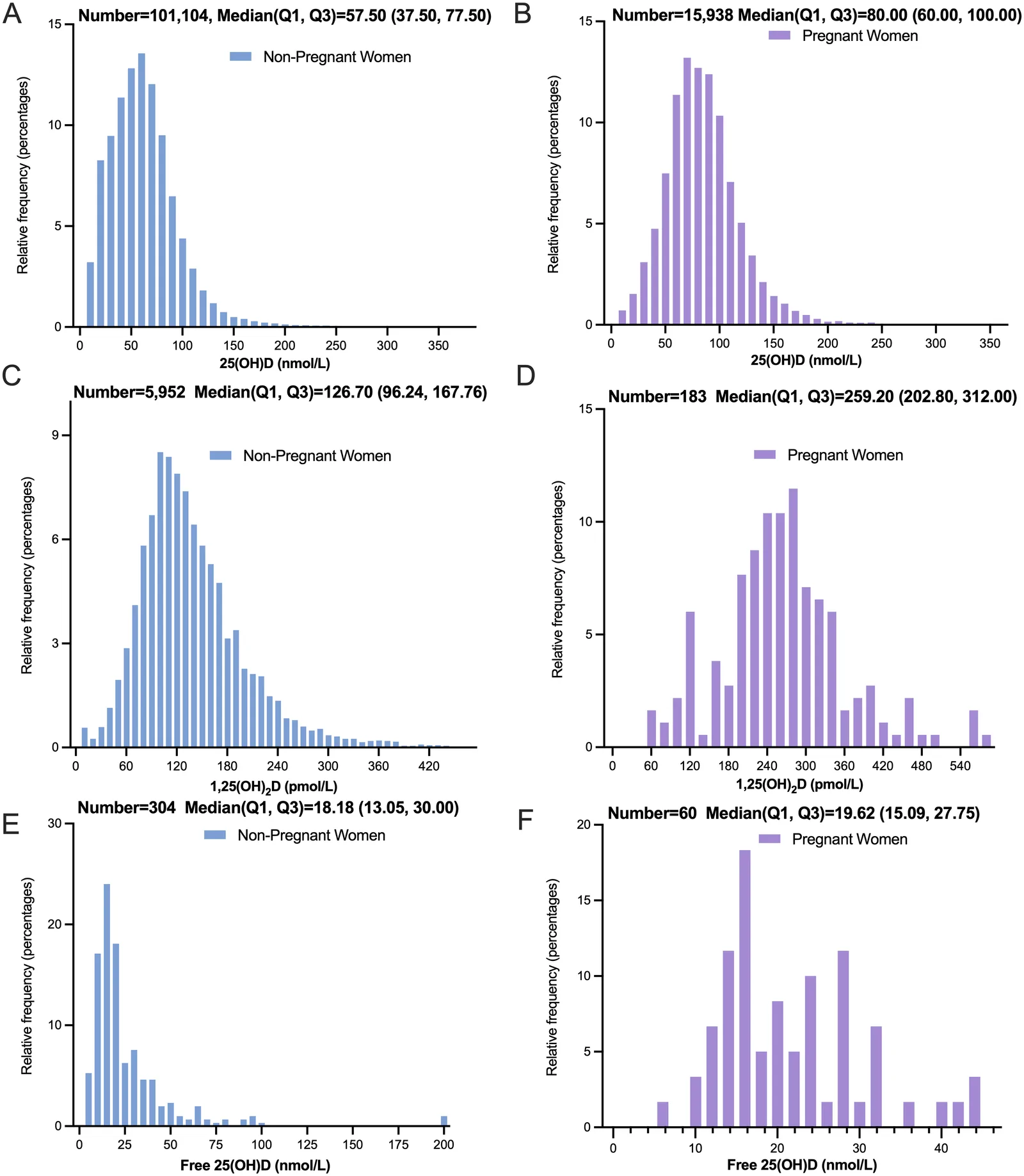
Distribution of serum vitamin D metabolites in pregnant and nonpregnant women. **(A)** Distribution of serum 25-hydroxyvitamin D [25(OH)D] concentrations in nonpregnant women. **(B)** Distribution of serum 25(OH)D concentrations in pregnant women. **(C)** Distribution of serum 1,25-dihydroxyvitamin D [1,25(OH)_2_D] concentrations in nonpregnant women. **(D)** Distribution of serum 1,25(OH)_2_D concentrations in pregnant women. **(E)** Distribution of free 25(OH)D concentrations in nonpregnant women. **(F)** Distribution of free 25(OH)D concentrations in pregnant women. Histograms represent relative frequency (%). Sample size (*n*), Median, and Interquartile Range (Q1, Q3) are annotated within each panel.

Circulating vitamin D metabolite concentrations differed markedly between pregnant and nonpregnant women (**Figure 3**). Median total 25(OH)D concentrations were significantly higher in pregnant women than in nonpregnant controls (**Figure 3A**, P < 0.0001). Similarly, total 1,25(OH)_2_D concentrations were substantially higher in pregnant than in nonpregnant women (**Figure 3B**, P < 0.0001). In contrast, no statistically significant difference in free 25(OH)D concentrations was detected between groups (**Figure 3C**).

**Figure 3:**
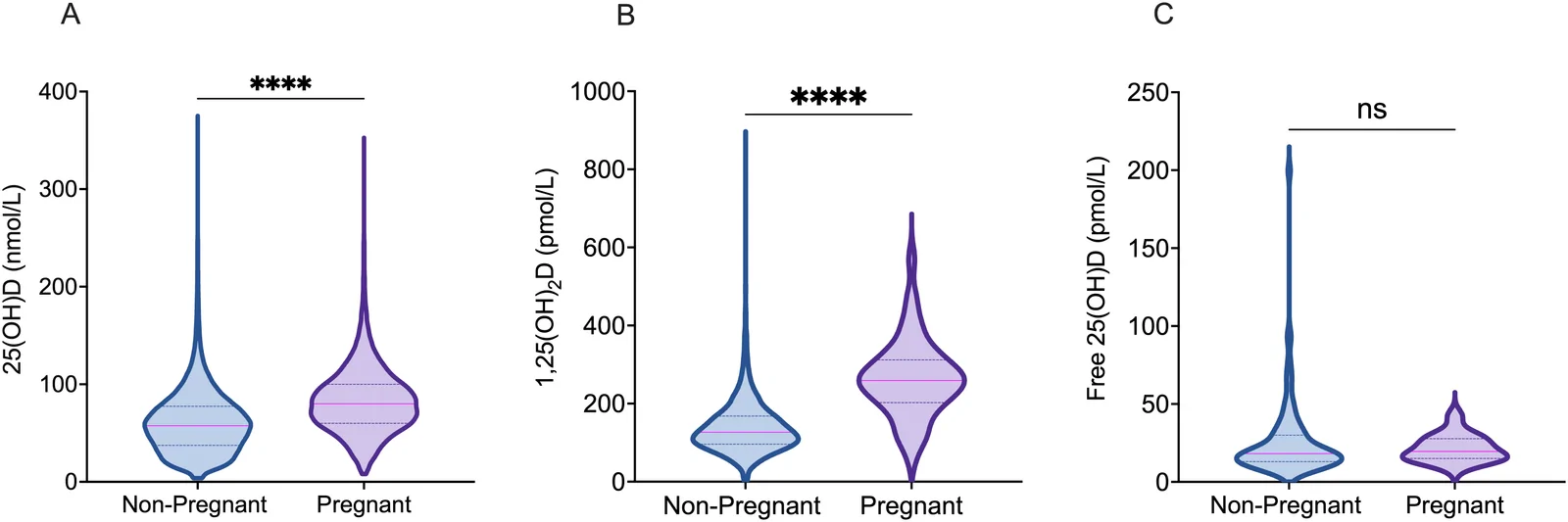
Comparison of circulating vitamin D metabolite concentrations between nonpregnant and pregnant women. Violin plots depict the distribution density and summary statistics for **(A)** serum 25(OH)D, **(B)** serum 1,25(OH)_2_D, and **(C)** serum free 25(OH)D concentrations. The width of each violin represents the probability density of the data at different concentration values. Solid horizontal lines indicate medians, and dotted lines denote interquartile ranges (25th–75th percentiles). Comparisons between pregnant and nonpregnant women were performed using the Mann–Whitney U test. ****P < 0.0001 versus the nonpregnant group; ns, not significant.

In age-adjusted linear regression analyses, pregnancy status was associated with higher circulating total 25(OH)D and 1,25(OH)_2_D concentrations (**Table 1**). Compared with nonpregnant women, pregnancy was associated with an increase of 22.07 nmol/L in total 25(OH)D (95% CI 21.50, 22.63; P < 0.001) and an increase of 125.20 pmol/L in 1,25(OH)_2_D (95% CI 115.39, 135.02; P < 0.001). In contrast, pregnancy status was not associated with free 25(OH)D concentrations after adjustment for age (β = -2.63 pmol/L; 95% CI -8.99, 3.74; P = 0.417). Age showed a modest positive association with free 25(OH)D but not with 1,25(OH)_2_D. Sensitivity analyses using log-transformed outcomes yielded conclusions consistent with the primary models: pregnancy remained associated with higher total 25(OH)D and total 1,25(OH)_2_D concentrations, whereas no statistically significant association was observed for free 25(OH)D (**Supplementary Table S10**). Regression diagnostics for the log-transformed models indicated statistically significant heteroscedasticity and identified some observations with relatively large studentized residuals; however, Cook’s distance values did not suggest that the fitted estimates were dominated by a single observation (**Supplementary Table S11**). Applying the same high-value exclusion threshold to both groups yielded a similar estimate for total 1,25(OH)_2_D. In the primary analysis, in which all observed concentrations were retained irrespective of pregnancy status, the age-adjusted difference was 125.20 pmol/L (95% CI 115.39, 135.02; P < 0.001). When concentrations >480 pmol/L were treated as missing in both pregnant and nonpregnant women, the corresponding difference was 117.77 pmol/L (95% CI 108.32, 127.22; P < 0.001) (**Supplementary Table S4**). In repeated equal-size random subsampling analyses, the estimated pregnancy-associated differences were similar to those obtained in the primary analyses (**Supplementary Table S6**). Across 1,000 iterations, the mean β estimates were 22.36 nmol/L for total 25(OH)D, 126.43 pmol/L for total 1,25(OH)_2_D, and -2.64 pmol/L for free 25(OH)D, compared with 22.07, 125.20, and -2.63, respectively, in the main analyses. Equalizing group sizes therefore did not materially alter the pregnancy-associated estimates.

**Table 1:** Age-adjusted multivariable linear regression analyses of circulating vitamin D metabolites.

Group	Variable	*n*	β	95% CI	*P*
Total 25(OH)D	Group				
	Nonpregnant Women	101,104	–	–	–
	Pregnant Women	15,938	22.07	21.50, 22.63	< 0.001
	Age	117,042	0.43	0.41, 0.46	< 0.001
Total 1,25(OH)_2_D	Group				
	Nonpregnant Women	5,952	–	–	–
	Pregnant Women	183	125.20	115.39, 135.02	< 0.001
	Age	6,135	-0.16	-0.37, 0.05	0.145
Free 25(OH)D	Group				
	Nonpregnant Women	304	–	–	–
	Pregnant Women	60	-2.63	-8.99, 3.74	0.417
	Age	364	0.49	0.17, 0.82	0.003

CI, Confidence Interval.

*β* coefficients represent the estimated difference in metabolite concentration associated with pregnancy status compared with nonpregnant women.

Additional analyses were performed to assess the comparability of participants with and without specialized vitamin D metabolite measurements (**Supplementary Table S7**). Among pregnant women, those with and without total 1,25(OH)_2_D measurements were similar in age, whereas gestational age differed between the groups (absolute SMD = 0.375). For free 25(OH)D, larger differences were observed in total 25(OH)D concentrations between participants with and without available measurements in both pregnant and nonpregnant women (absolute SMDs = 0.415 and 0.605, respectively). Overall, some available characteristics differed between the analyte-specific subcohorts and participants without the corresponding measurements, particularly for free 25(OH)D.

Gestational age–related patterns differed between the two vitamin D metabolites (**Figure 4**). For 25(OH)D, gestational age was associated with serum concentrations in a nonlinear manner after adjustment for maternal age (overall association, *P* < 0.0001; test for nonlinearity, *P* < 0.0001). Predicted total 25(OH)D concentrations showed a nonlinear cross-sectional pattern across gestational age, with lower values after the earliest gestational weeks, higher values at later gestational ages, and an apparent plateau in late gestation.

**Figure 4:**
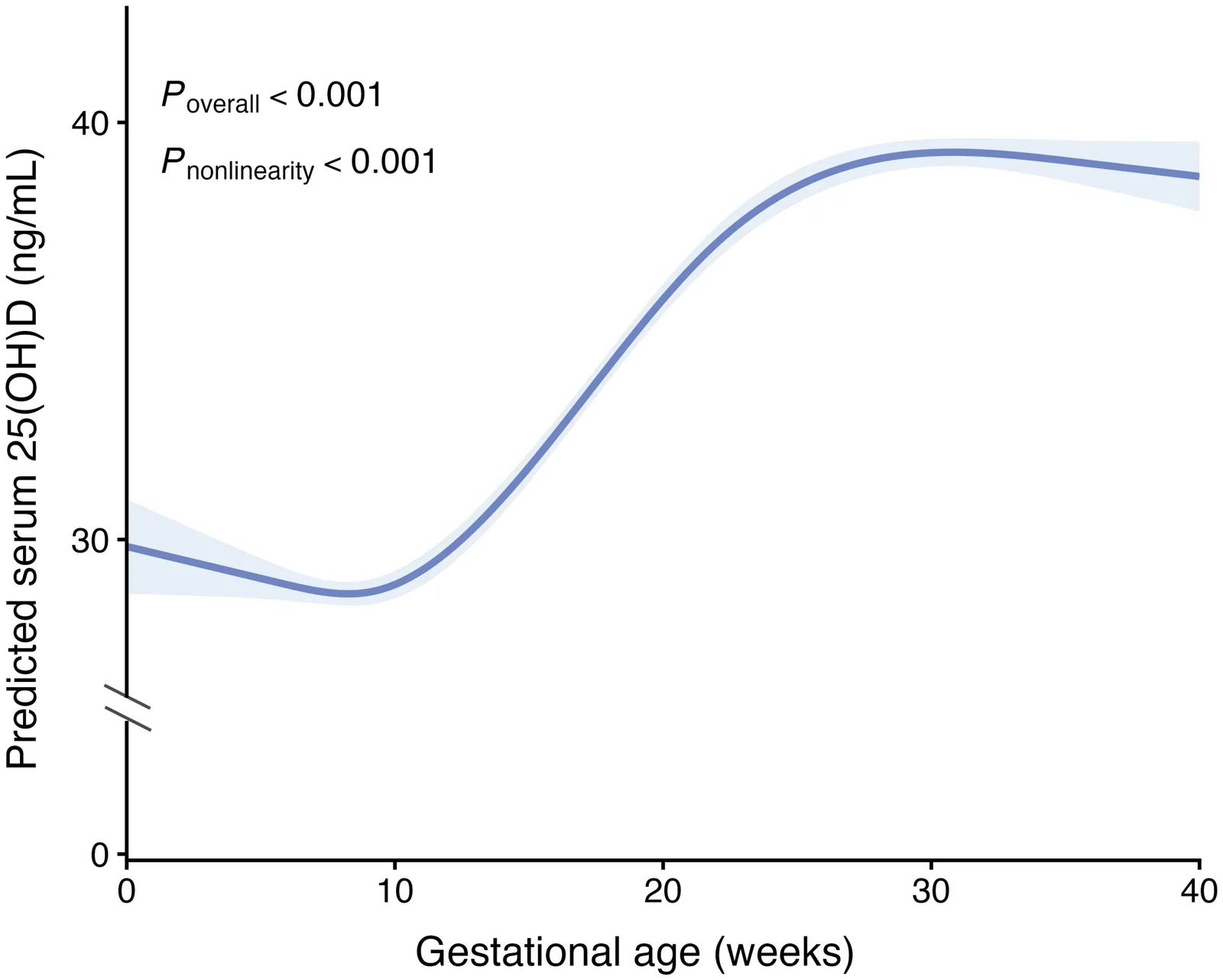
Association between gestational age and serum 25(OH)D concentrations during pregnancy. Restricted cubic spline showing the association between gestational week and predicted serum 25(OH)D concentrations, adjusted for maternal age. Shaded areas indicate 95% CIs. A break is shown in the y-axis to improve visualization.

In contrast, serum 1,25(OH)_2_D concentrations were positively associated with gestational age in an approximately linear manner after adjustment for maternal age. Each 1-week increase in gestational age was associated with a 4 pmol/L higher serum 1,25(OH)_2_D concentration (95% CI 2.59, 5.4; *P* < 0.001) (**Figure 5**).

**Figure 5:**
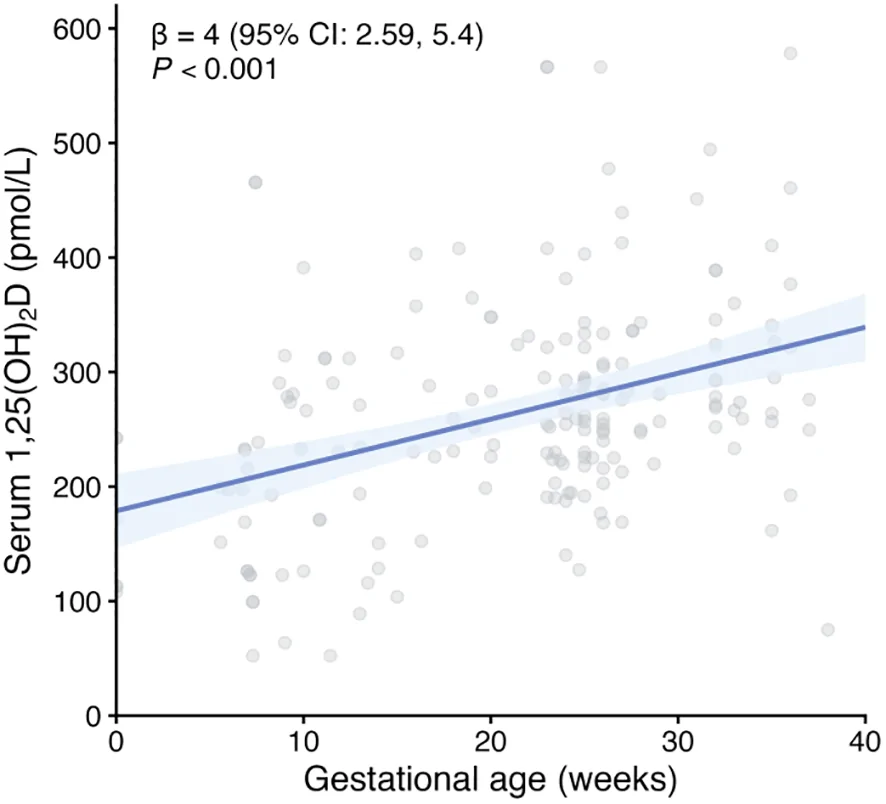
Association between gestational age and serum 1,25(OH)_2_D concentrations during pregnancy. Scatterplot with fitted linear regression line showing a positive association between gestational week and serum 1,25(OH)_2_D concentrations. Shaded areas represent 95% CIs.

To characterize gestational-stage differences in vitamin D metabolite concentrations relative to the nonpregnant reference group, age-adjusted linear regression analyses were performed by gestational trimester (**Table 2**). Compared with nonpregnant women, serum 25(OH)D concentrations were already higher in the first trimester (*β* = 10.60 nmol/L; 95% CI 9.83, 11.37; *P* < 0.001). The between-group difference was larger in the second trimester (*β* = 30.08 nmol/L; 95% CI 29.09, 31.07) and was greatest in the third trimester (*β* = 37.40 nmol/L; 95% CI 36.27, 38.53). Accordingly, median total 25(OH)D concentrations were 67.50 nmol/L in the first trimester and 90.00 nmol/L in the third trimester, compared with 57.50 nmol/L in nonpregnant women.

**Table 2:** Age-adjusted associations of gestational trimesters with serum vitamin D metabolites compared to the general population.

Variable	Group	*n*	Median [IQR]	Age-adjusted beta (95% CI)	*P*
Total 25(OH)D	Nonpregnant Women	101,104	57.5 [37.5, 77.5]	0 (Reference)	–
	1st Trimester (Early)	7,871	67.5 [52.5, 85]	10.60 (9.83, 11.37)	< 0.001
	2nd Trimester (Mid)	4,595	90 [72.5, 107.5]	30.08 (29.09, 31.07)	< 0.001
	3rd Trimester (Late)	3,472	95 [77.5, 117.5]	37.40 (36.27, 38.53)	< 0.001
Total 1,25(OH)_2_D	Nonpregnant Women	5,952	126.72 [96.24, 168.00]	0 (Reference)	–
	1st Trimester (Early)	49	197.52 [122.88, 271.20]	67.37 (48.69, 86.04)	< 0.001
	2nd Trimester (Mid)	96	260.40 [224.76, 321.60]	137.99 (124.60, 151.39)	< 0.001
	3rd Trimester (Late)	38	279.60 [256.80, 345.00]	167.42 (146.24, 188.61)	< 0.001
Free 25(OH)D	Nonpregnant Women	304	18.2 [13, 30]	0 (Reference)	
	1st Trimester (Early)	34	20.7 [14.7, 28.3]	-1.68 (-9.84, 6.48)	0.686
	2nd Trimester (Mid)	15	19.4 [16.1, 22.9]	-4.08 (-15.91, 7.75)	0.498
	3rd Trimester (Late)	11	16.5 [13.9, 27.3]	-3.54 (-17.21, 10.14)	0.611

Values are presented as median (interquartile range, IQR).

Age-adjusted β coefficients and 95% confidence intervals (CIs) were estimated using linear regression models, with nonpregnant women as the reference group.

Serum total 1,25(OH)_2_D concentrations were significantly higher in pregnant women than in nonpregnant women beginning in the first trimester (*β* = 67.37 pmol/L; 95% CI 48.69, 86.04; *P* < 0.001), with further increases observed in the second trimester (*β* = 137.99 pmol/L; 95% CI 124.60, 151.39) and the largest differences in the third trimester (*β* = 167.42 pmol/L; 95% CI 146.24, 188.61). The median total 1,25(OH)_2_D concentration was 279.60 pmol/L in the third trimester, compared with 126.72 pmol/L in nonpregnant women.

By contrast, free 25(OH)D concentrations did not differ significantly from those in nonpregnant women in any trimester. The age-adjusted β coefficients were -1.68 (95% CI -9.84, 6.48; *P* = 0.686) in the first trimester, -4.08 (95% CI-15.91, 7.75; *P* = 0.498) in the second trimester, and -3.54 (95% CI -17.21, 10.14; *P* = 0.611) in the third trimester.

Among participants with valid measurements of both total 25(OH)D and total 1,25(OH)_2_D, the median molar 25(OH)D/1,25(OH)_2_D ratio was lower in pregnant than in nonpregnant women (289.80 [IQR 210.9–394.5] vs 392.9 [IQR 260.4–575.6]). In the age-adjusted log-transformed model, pregnancy was associated with a lower ratio (adjusted ratio 0.754, 95% CI 0.684–0.831). The ratio remained lower than the nonpregnant reference across all three trimesters, with similar findings in the sensitivity analysis (**Supplementary Table S8**).

### 3.2 Meta-analysis

For total 1,25(OH)_2_D, 11 studies, including the present study, were eligible for quantitative synthesis, of which nine were included in the primary meta-analysis. For free 25(OH)D, three studies were eligible for meta-analysis. The selection process is provided in **Supplementary Figure S1**.

**Supplementary Table S1** summarises published studies of total 1,25(OH)_2_D in pregnancy. Despite considerable methodological heterogeneity across assay platforms, most studies reported higher circulating total 1,25(OH)_2_D concentrations in pregnant than in nonpregnant women and/or increases with advancing gestation. Notably, Møller et al. (29) reported lower baseline levels in pregnant cohorts, underscoring that population characteristics, gestational timing, and assay selection may influence between-group contrasts.

The meta-analysis forest plot (**Figure 6**) for circulating total 1,25(OH)_2_D showed higher circulating concentrations in pregnancy than in nonpregnant controls across all included studies (4, 5, 30–35) with study-specific ratios of means ranging from 1.33 to 2.36. The random-effects pooled estimate indicated approximately 1.9-fold higher total 1,25(OH)_2_D concentrations in pregnant than in nonpregnant women (ROM = 1.88; 95% CI, 1.67–2.12). After exclusion of the present cohort, the pooled estimate was essentially unchanged (ROM = 1.87; 95% CI 1.62–2.16; 8 studies), indicating that the pooled estimate was not materially influenced by inclusion of the present cohort (**Supplementary Table S12**). Heterogeneity was considerable (I² = 76.63%), but sensitivity analyses supported robustness and Egger’s test did not provide evidence of small-study effects.

**Figure 6:**
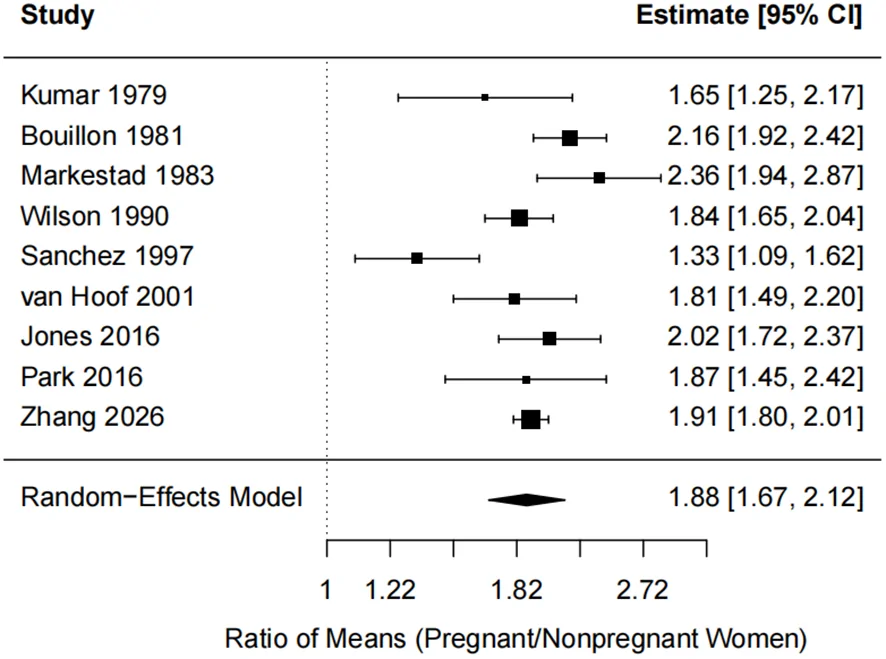
Forest plot for the primary meta-analysis of total 1,25-dihydroxyvitamin D. The pooled ratio of means (ROM; Preg/Ctrl) from the random-effects model was 1.88 (95% CI 1.67 to 2.12). Between-study heterogeneity was considerable (I² = 76.63%, *τ*² = 0.00156, *P* for heterogeneity = 0.0021). All included studies showed ROM estimates greater than 1, indicating consistently higher total 1,25(OH)_2_D concentrations in pregnant women than in nonpregnant controls, although the magnitude of the association varied across studies.

**Supplementary Table S2** summarises published studies of free or bioavailable 25(OH)D, assessed either indirectly through calculation using total 25(OH)D, VDBP, and albumin or by direct enzyme-linked immunosorbent assay. Most studies reported lower free 25(OH)D concentrations during pregnancy or declines across gestation, although findings were heterogeneous, with some cohorts showing U-shaped trajectories or relatively stable concentrations. Methodological discrepancies between directly measured and calculated free 25(OH)D indicate substantial sensitivity to the analytical approach used. Differences between these approaches may be particularly relevant during pregnancy because of pregnancy-associated changes in VDBP concentration and binding characteristics.

For free 25(OH)D, random-effects meta-analysis (34, 35) (**Figure 7**) of three studies suggested modestly lower concentrations in pregnant than in nonpregnant women (pooled ROM = 0.78; 95% CI 0.63, 0.97; I² = 17.37%). After exclusion of the present cohort, the pooled ROM remained below 1 (0.74), but the 95% CI widened substantially and crossed 1 (0.41–1.33; 2 studies), indicating substantial imprecision in the estimate based solely on the independent published studies (**Supplementary Table S12**). The small number of studies limits precision, and analytical differences between measured and calculated fractions remain important.

**Figure 7:**
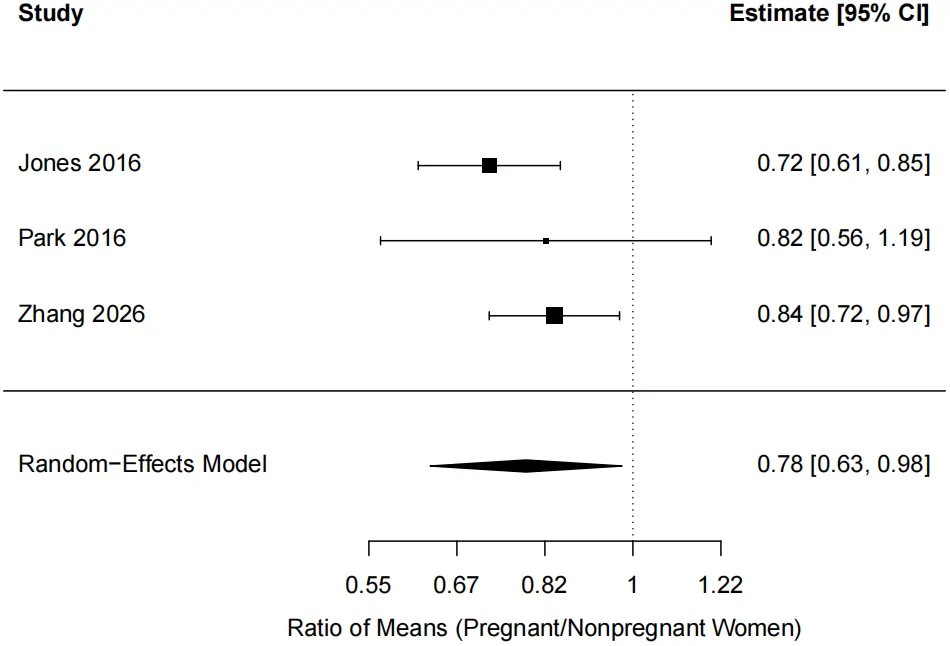
Forest plot for the primary meta-analysis of free 25-hydroxyvitamin D. The pooled ratio of means (ROM; Preg/Ctrl) from the random-effects model was 0.78 (95% CI 0.63 to 0.97). Between-study heterogeneity was low (I² = 17.37%, *τ*² = 0.0021, *P* for heterogeneity = 0.41). Leave-one-out analysis suggested that the overall direction of the association was unchanged after omitting any single study, although the magnitude of the pooled estimate varied slightly because only three studies were included.

## 4 Discussion

### 4.1 Main findings and interpretation

In this real-world laboratory study of women of reproductive age, pregnancy was associated with higher total 25(OH)D and markedly higher total 1,25(OH)_2_D concentrations, whereas free 25(OH)D did not differ significantly between pregnant and nonpregnant women in the available subgroup. In the cross-sectional gestational-age analyses, total 25(OH)D and total 1,25(OH)_2_D were higher at later gestational ages, whereas no clear gestational pattern could be established for free 25(OH)D. Complementary meta-analyses showed substantially higher total 1,25(OH)_2_D during pregnancy and suggested modestly lower free 25(OH)D concentrations; sensitivity analyses excluding the present cohort yielded a similar pooled pattern for total 1,25(OH)_2_D but substantially greater uncertainty for free 25(OH)D. Together, these findings indicate differing pregnancy-associated patterns across circulating vitamin D metabolites, although the evidence for free 25(OH)D remains limited. Previous studies were generally smaller, often restricted to a single gestational period, and heterogeneous in assay methodology. The present study provides high statistical precision for total 25(OH)D and additional information on total 1,25(OH)_2_D across gestational ages, although the direct free 25(OH)D findings remain exploratory. The three metabolites were assessed in separate analyte-specific subcohorts, with no participant having valid measurements of all three simultaneously; therefore, differences in their pregnancy-associated patterns should not be interpreted as coordinated within-participant changes. A strength of the study is that these biologically distinct vitamin D measures were evaluated within a common routine-care laboratory framework.

### 4.2 Interpretation of individual vitamin D metabolites

The markedly higher circulating total 1,25(OH)_2_D concentrations observed in pregnant women are consistent with classical physiological studies and more recent longitudinal investigations showing that calcitriol rises early in pregnancy and remains elevated until delivery (1, 4–6, 29, 36, 37). This adaptation supports increased intestinal calcium absorption and maternal–fetal calcium transfer (37). It reflects enhanced renal and extrarenal vitamin D metabolism, while the placenta also possesses the capacity to take up and metabolize 25(OH)D within the fetoplacental unit (8, 37, 38). In the present study, total 1,25(OH)_2_D showed a graded cross-sectional association with gestational age among women with available measurements. Total 25(OH)D and total 1,25(OH)_2_D concentrations were positively associated in both pregnant and nonpregnant women, with a stronger concentration–concentration relationship observed during pregnancy (**Supplementary Figure S2**). Complementing this finding, the molar total 25(OH)D/total 1,25(OH)_2_D ratio was lower during pregnancy and remained below the nonpregnant reference across all three trimesters (**Supplementary Table S8**). Together, these findings are compatible with greater relative representation of the active metabolite in the circulation during pregnancy. However, the substrate-to-product ratio should be regarded as an indirect metabolic index rather than a direct measure of CYP27B1 activity, because circulating concentrations are also influenced by substrate availability, binding, clearance, and renal and extrarenal metabolism. All study-specific estimates in the meta-analysis were above unity, and the pooled ratio of means indicated an approximately 1.9-fold higher total 1,25(OH)_2_D concentrations in pregnant than in nonpregnant women. Importantly, exclusion of the present cohort from the meta-analysis yielded an almost identical pooled estimate (ROM 1.87, 95% CI 1.62–2.16), indicating that the pooled elevation was not dependent on inclusion of the present cohort. The substantial between-study heterogeneity likely reflects differences in assay platform, gestational timing, substrate availability, supplementation, renal function, and population characteristics. Accordingly, variation in the magnitude of the between-group difference should not be interpreted as evidence against gestational calcitriol upregulation but rather highlights the need for assay- and gestation-specific interpretation.

The higher total 25(OH)D concentrations observed in pregnant women require more cautious interpretation. Pregnancy may be accompanied by increases in total 25(OH)D, VDBP, total 1,25(OH)_2_D, and other vitamin D metabolites even when vitamin D intake is comparable between pregnant and nonpregnant women (34, 35). Longitudinal studies have reported variable total 25(OH)D trajectories across pregnancy, including gestational and season-dependent changes (39, 40). In the present cohort, total 25(OH)D showed a nonlinear cross-sectional pattern across gestational age, with lower predicted concentrations after the earliest gestational weeks, higher concentrations at later gestational ages, and an apparent late-gestational plateau. This pattern may reflect estrogen-driven increases in VDBP and altered vitamin D kinetics but may also be influenced by supplementation, season, lifestyle, and clinical indication for testing (37, 39–41). Because these determinants were unavailable, the observed trajectory should be interpreted as a real-world biochemical pattern rather than definitive evidence that pregnancy itself increases maternal vitamin D stores or tissue-available vitamin D.

The relationship between total and free 25(OH)D is biologically plausible because the gestational rise in VDBP expands the protein-bound circulating pool without necessarily increasing the free fraction (9, 10, 34, 41). In the small subgroup with directly measured free 25(OH)D, no statistically significant difference was detected between pregnant and nonpregnant women or across trimesters. However, only 60 pregnant women had available free 25(OH)D measurements, and trimester-specific sample sizes were particularly small, including only 11 women in the third trimester. These analyses therefore have limited statistical precision and cannot establish either equivalence between pregnant and nonpregnant women or physiological stability across gestation. By contrast, the primary meta-analysis suggested an approximately 22% lower concentration during pregnancy. When the present cohort was excluded, the pooled estimate remained below unity (ROM 0.74), but the 95% CI widened and crossed unity (0.41–1.33). Studies directly comparing free 25(OH)D between pregnant and nonpregnant women are scarce; after exclusion of the present cohort, only two independent studies remained, both of which used calculated rather than directly measured free 25(OH)D. This distinction is important because calculated and directly measured free 25(OH)D are not analytically interchangeable (9, 25, 42, 43). Published studies have not been entirely consistent, and stable, lower, or higher absolute free 25(OH)D concentrations have been reported depending on gestational timing, population characteristics, supplementation, and measurement method. Taken together, the available evidence does not permit a firm conclusion regarding or magnitude of pregnancy-associated differences in free 25(OH)D. Larger, adequately powered longitudinal studies with repeated measurements across pregnancy are needed to characterize within-person gestational changes.

More broadly, circulating vitamin D concentrations should be distinguished from biological availability and tissue-level activity. Pregnancy-associated increases in VDBP can expand the protein-bound circulating pool and alter the relationship between total and free vitamin D concentrations; consequently, higher total 25(OH)D does not necessarily imply a proportional increase in circulating free 25(OH)D. Biological effects also depend on cellular uptake, local activation and inactivation of vitamin D metabolites, and subsequent VDR-mediated signaling. Although 1,25(OH)_2_D is the principal ligand for VDR activation, higher circulating total 1,25(OH)_2_D concentrations do not by themselves establish proportionally greater tissue-level signaling or biological effects. These distinctions are particularly relevant during pregnancy, when changes in binding proteins and vitamin D metabolism may alter circulating metabolite concentrations without directly reflecting tissue-level vitamin D activity.

### 4.3 Clinical and analytical implications

Assay methodology is important when interpreting these findings. Total 25(OH)D, free 25(OH)D, and total 1,25(OH)_2_D were measured using different immunoassay platforms, and absolute concentrations are method-dependent. Immunoassay results may differ from LC-MS/MS because of calibration, metabolite cross-reactivity, release from VDBP, and pregnancy-related matrix effects (44, 45). Measurement of total 1,25(OH)_2_D is particularly challenging because of its low circulating concentration and method-specific analytical limitations (46, 47). Directly measured and calculated free 25(OH)D concentrations are also not analytically interchangeable (9, 25, 42, 43). Any pregnancy-specific reference intervals should therefore be assay-specific, and serial measurements should preferably be performed using the same validated platform.

These pregnancy-associated patterns have implications primarily for laboratory interpretation and reference interval development. Because total 1,25(OH)_2_D concentrations are physiologically higher during pregnancy, application of nonpregnant reference intervals may classify physiologically expected gestational concentrations as abnormally high. Conversely, a total 1,25(OH)_2_D concentration within or below a conventional nonpregnant reference interval may be lower than expected for the corresponding gestational stage. However, the clinical significance of such a result remains uncertain, and total 1,25(OH)_2_D should not be used as a routine marker of vitamin D nutritional status. When measurement is clinically indicated, results should be interpreted in the context of the indication for testing, calcium, phosphate, parathyroid hormone, renal function, 25(OH)D, gestational age, and the analytical method (8, 37). Direct measurement of free 25(OH)D has been proposed as potentially informative in selected cases of suspected altered binding-protein physiology or unexplained discordance between total 25(OH)D and the clinical or biochemical phenotype. However, its added clinical value, analytical standardization, and decision thresholds during pregnancy remain unestablished (9–11, 25, 42, 43).

Current guidance suggests empiric vitamin D supplementation during pregnancy and advises against routine biochemical testing in generally healthy pregnant individuals without an established indication (19, 48). The present findings, together with previous physiological evidence, highlight that pregnancy changes the biochemical context in which vitamin D results are interpreted and support further investigation of gestation- and assay-specific reference frameworks. Such frameworks require prospective validation against clinically meaningful maternal, placental, fetal, and neonatal outcomes before they can guide clinical decision-making.

### 4.4 Strengths and limitations

This study has several strengths. It draws on a large real-world laboratory dataset and provides particularly high statistical precision for total 25(OH)D. It also enabled evaluation of total 1,25(OH)_2_D and directly measured free 25(OH)D in analyte-specific subcohorts, although sample sizes differed substantially among analytes. Gestational patterns were examined both categorically and continuously for analytes with sufficient available data. Analytical robustness was assessed using age-adjusted models, nonlinear analyses, log-transformed sensitivity analyses, and analyte-specific sensitivity analyses for high-value handling. The complementary systematic review and meta-analysis also allowed the cohort findings to be interpreted in the context of evidence from different populations and analytical platforms. In addition, each analyte was measured within a single clinical laboratory using a consistent platform, reducing within-cohort interlaboratory variability.

Several limitations should also be considered. The cohort was laboratory-based and indication-driven rather than population-based and may therefore overrepresent women with clinical indications for vitamin D or mineral-metabolism testing. Accordingly, the observed distributions should not be interpreted as representative population distributions and may not be fully generalizable to all pregnant or nonpregnant women of reproductive age. Because of the cross-sectional design, gestational patterns represent between-person differences rather than within-person changes, and associations with gestational age should therefore not be interpreted as demonstrating temporal increases within individuals during pregnancy. Maternal age was the only covariate available in a sufficiently complete and standardized form for adjustment in pregnancy-status models, while information on supplementation, season, body mass index, ethnicity, lifestyle factors, renal and hepatic function, mineral metabolism, comorbidities, and pregnancy complications was unavailable or incomplete; residual confounding therefore cannot be excluded. VDBP and albumin measurements were also unavailable, precluding calculation of free 25(OH)D and direct comparison between calculated and measured free 25(OH)D within the cohort. Consequently, the contribution of pregnancy-related changes in VDBP concentration or binding affinity to the observed relationship between total and free 25(OH)D could not be evaluated directly.

Analyte availability differed markedly, particularly for total 1,25(OH)_2_D and free 25(OH)D. Although repeated equal-size random subsampling yielded central estimates similar to those from the main analyses, suggesting that the results were not highly sensitive to unequal group sizes, this approach cannot address indication-related selection into testing. The total 1,25(OH)_2_D and particularly the free 25(OH)D subcohorts were substantially smaller than the total 25(OH)D cohort and were subject to indication-related selection, while the indications for ordering these specialized assays were unavailable. Because no participant had valid measurements of all three metabolites simultaneously, coordinated within-participant differences among metabolites could not be assessed. The particularly small free 25(OH)D subgroup also limited precision, precluding interpretation of the nonsignificant result as evidence of equivalence. In addition, measurements were assay-specific and were not validated in parallel against reference procedures, so absolute concentrations may not be transferable across analytical platforms. Clinical outcome data were unavailable, preventing assessment of associations between individual vitamin D metabolites and maternal or pregnancy outcomes or comparison of their relative predictive performance. Finally, the meta-analyses included relatively few and methodologically heterogeneous studies, particularly for free 25(OH)D. Because the present cohort also contributed to the primary quantitative synthesis, the cohort and primary pooled findings should not be regarded as fully independent lines of evidence. Sensitivity analyses excluding the present cohort partly addressed this concern: the pooled estimate for total 1,25(OH)_2_D remained essentially unchanged, whereas the free 25(OH)D estimate became substantially less precise and its confidence interval crossed unity.

### 4.5 Future implications and perspectives

Future research should prioritize prospective longitudinal cohorts with repeated sampling before conception, throughout pregnancy, and postpartum. Such studies should combine standardized measurements of total and free vitamin D metabolites with assessment of VDBP, albumin, calcium–phosphate metabolism, kidney function, supplementation, season, body mass index, ethnicity, lifestyle factors, comorbidities, and pregnancy complications.

Direct and calculated free 25(OH)D should be compared with reference approaches, and proposed gestation-specific reference intervals should be developed separately for validated analytical platforms. Future studies should also determine whether free 25(OH)D, bioavailable 25(OH)D, the free-to-total ratio, or composite indices incorporating VDBP improve prediction of maternal and neonatal outcomes beyond total 25(OH)D alone. Studies evaluating “bioavailable” vitamin D should explicitly define the calculation method and acknowledge that albumin-bound and VDBP-bound metabolites may not be biologically equivalent across tissues. Physiological reference intervals and clinical decision limits should remain conceptually distinct, because decision limits require evidence linking biomarker concentrations to clinically meaningful outcomes and demonstrating benefit from biomarker-guided intervention.

## 5 Conclusion

This study provides large-scale real-world and meta-analytic evidence that circulating vitamin D metabolites show distinct pregnancy-associated patterns. The markedly higher total 1,25(OH)_2_D concentrations in pregnant women and their positive cross-sectional association with gestational age are consistent with established physiological adaptations of pregnancy. Higher total 25(OH)D concentrations were also observed during pregnancy; however, these findings should be interpreted as associations rather than as evidence that pregnancy itself increases maternal vitamin D stores. In contrast, no statistically significant difference in free 25(OH)D was detected in the much smaller available subgroup; this finding should be considered exploratory and requires confirmation in larger, adequately powered studies. Prospective longitudinal studies using standardized assays, comprehensive assessment of VDBP and mineral metabolism, and clinically relevant maternal and neonatal outcomes are required before gestation-specific decision limits or alternative biomarkers can be recommended.

## Data Availability

The raw data supporting the conclusions of this article will be made available by the authors, without undue reservation.
