# Supplementary material for: Pregnancy-associated alterations in circulating vitamin D metabolites: higher total 25-hydroxyvitamin D and 1,25-dihydroxyvitamin D without increased free 25-hydroxyvitamin D
Source: Front Endocrinol (Lausanne). 2026 Oct 22;17:1957418. doi: 10.3389/fendo.2026.1957418 (PMC13735605; doi:10.3389/fendo.2026.1957418)
Supplement: Supplementary file 1 [file DataSheet1.docx]

**Content**

[Supplementary Methods:	2](#_Toc66306301)

[Search strategy:	2](#_Toc2013471761)

[Supplementary Figure S1. Study screening flow diagram.	4](#_Toc372577701)

[Supplementary Figure S2. Association between serum 25-hydroxyvitamin D and 1,25-dihydroxyvitamin D concentrations by pregnancy status.	4](#_Toc1998589702)

[Supplementary Figure S3. Funnel plot for the meta-analysis of studies assessing circulating 1,25-dihydroxyvitamin D concentrations in pregnant versus nonpregnant control participants.	4](#_Toc1505398787)

[Supplementary Figure S4. Influence diagnostics for the primary meta-analysis of 1,25-dihydroxyvitamin D.	5](#_Toc1732567802)

[Supplementary Table S1. Total 1,25-dihydroxyvitamin D and Pregnancy: Study Classification	5](#_Toc1536278541)

[Supplementary Table S2. Free 25-hydroxyvitamin D and Pregnancy: Study Classification	9](#_Toc1037550706)

[Supplementary Table S3. Sensitivity analysis of high-value handling for total 25-hydroxyvitamin D.	12](#_Toc547502102)

[Supplementary Table S4. Sensitivity analysis excluding total 1,25-dihydroxyvitamin D concentrations >480 pmol/L in both groups.	12](#_Toc2047884566)

[Supplementary Table S5. Sensitivity analysis of extreme-value handling for free 25-hydroxyvitamin D.	12](#_Toc1075490293)

[Supplementary Table S6 . Main and repeated equal-size random subsampling analyses of pregnancy-associated differences in circulating vitamin D metabolites	13](#_Toc395497652)

[Supplementary Table S7. Comparison of available characteristics between participants with and without specialized vitamin D metabolite measurements	13](#_Toc667149699)

[Supplementary Table S8. Primary and sensitivity analyses of the molar ratio of total 25(OH)D to total 1,25(OH)](#_Toc772870106)_[2](#_Toc772870106)_[D according to pregnancy status and gestational trimester	13](#_Toc772870106)

[Supplementary Table S9. Available participant characteristics and analyte-specific vitamin D measurements according to pregnancy status	14](#_Toc1646774486)

[Supplementary Table S10. Primary and log-transformed sensitivity analyses for the main age-adjusted regression models.	14](#_Toc569543666)

[Supplementary Table S11. Regression diagnostics for the log-transformed age-adjusted models	14](#_Toc985779783)

[Supplementary Table S12. Primary and sensitivity meta-analyses of vitamin D metabolites in pregnant versus nonpregnant women	14](#_Toc164476276)

[Supplementary Table S13. JBI critical appraisal of analytical cross-sectional studies included in the meta-analysis.	15](#_Toc541317043)

[Supplementary Table S14. JBI critical appraisal of cohort studies included in the meta-analysis.	15](#_Toc1174813009)

[Supplementary References：	16](#_Toc1117591745)

# Supplementary Methods:

# Search strategy:

The principal search concepts and search terms are provided below. For each database, search terms and search functions were adapted to the database structure. Only studies published in English were considered. In addition to database searching, the reference lists of included studies were manually screened for additional eligible articles, and conference abstracts and prospective trial registries were searched to identify potentially relevant unpublished or ongoing studies.

The search strategy combined Medical Subject Headings (MeSH) and free-text terms related to vitamin D metabolites and pregnancy. The following search terms were used: (“free 25-hydroxyvitamin D” OR “free 25(OH)D” OR “bioavailable 25-hydroxyvitamin D” OR “bioavailable 25(OH)D” OR “vitamin D-binding protein” OR “VDBP”) AND (“pregnancy” OR “pregnant” OR “gestation” OR “trimester” OR “maternal”). In addition, terms related to active vitamin D metabolites were included: (“1,25-dihydroxyvitamin D” OR “1,25(OH)2D” OR “calcitriol”) AND (“pregnancy” OR “pregnant” OR “gestation” OR “maternal”).

### **Additional study selection details**

### Retrieved records were screened by title and abstract to assess potential relevance. Full texts were obtained for studies that appeared to meet eligibility criteria or for which eligibility could not be determined from the title and abstract alone. Full-text articles were then assessed against predefined inclusion and exclusion criteria. Duplicate records were removed before screening. When multiple publications reported data from the same study population, the most comprehensive or most recent report was retained for data extraction, while related publications were reviewed for supplementary information.

### If studies reported multiple physiological groups or mixed longitudinal and cross-sectional designs, only one prespecified independent comparison between pregnant and nonpregnant women was selected for the primary meta-analysis to avoid duplicate use of participants.

### **Unit conversion and data handling**

For manuscript reporting, total 25(OH)D concentrations were expressed in nmol/L and converted from ng/mL using the factor 1 ng/mL = 2.5 nmol/L. Total 1,25(OH)_2_D concentrations were expressed in pmol/L and converted from pg/mL using the factor 1 pg/mL = 2.4 pmol/L. Free 25(OH)D concentrations were expressed in pmol/L and converted from pg/mL using the factor 1 pg/mL = 2.5 pmol/L.

For the meta-analysis, metabolite concentrations were harmonized to these units before effect-size calculation. When studies reported multiple groups or gestational stages, means and measures of variability were extracted when available. If only medians and ranges or interquartile ranges were reported, the corresponding means and standard deviations were estimated using established statistical methods when necessary. When multiple publications originated from the same cohort, only one publication was included in the primary analysis.

### **Repeated equal-size random subsampling sensitivity analysis**

To further evaluate whether the marked imbalance in sample sizes between pregnant and nonpregnant groups influenced the estimated associations, repeated equal-size random subsampling was performed separately for each vitamin D metabolite. For each iteration, all pregnant women with an available measurement of the respective metabolite were retained, while an equal number of nonpregnant women were randomly sampled from the corresponding analyte-specific cohort. Thus, each iteration included 15,938 pregnant and 15,938 nonpregnant women for total 25(OH)D, 183 women per group for total 1,25(OH)_2_D, and 60 women per group for free 25(OH)D. Age matching was not imposed; instead, age was included as a covariate in each regression model, consistent with the main analysis.

For each metabolite, the random subsampling and age-adjusted linear regression procedure was repeated 1,000 times using a fixed random seed  to ensure reproducibility. The distribution of the pregnancy-status β coefficients across iterations was summarized by the mean β and the empirical 2.5th and 97.5th percentiles. These percentiles characterize the variability of effect estimates across repeated random subsamples and do not represent model-based 95% confidence intervals.

**Subgroup comparability analysis**
To evaluate whether participants with available specialized vitamin D metabolite measurements differed from the broader study population, participants with and without total 1,25(OH)_2_D measurements and those with and without free 25(OH)D measurements were compared separately within pregnant and nonpregnant groups. Measurement status was defined according to the availability of the original assay result before analyte-specific high-value handling. Available characteristics included age and total 25(OH)D concentrations, with gestational age additionally assessed among pregnant women. Continuous characteristics were summarized as medians (IQRs), and absolute standardized mean differences (SMDs), calculated using means and pooled standard deviations, were used to quantify between-group differences. Because clinical indications for ordering specialized vitamin D measurements were not available in a sufficiently standardized form, these analyses were intended to assess comparability on observed characteristics rather than to establish representativeness with respect to unmeasured clinical factors.

**Molar total 25(OH)D/total 1,25(OH)_2_D ratio analysis**
To further examine the relationship between vitamin D substrate and active metabolite, the molar ratio of total 25(OH)D to total 1,25(OH)_2_D was calculated among participants with valid measurements of both analytes. Because total 25(OH)D was expressed in nmol/L and total 1,25(OH)_2_D in pmol/L, total 25(OH)D concentrations were converted to pmol/L by multiplication by 1,000 before calculation of the ratio. The resulting ratio was summarized as median (IQR). Pregnancy-status and trimester-specific differences in the ratio were evaluated using age-adjusted regression models, with nonpregnant women as the reference group. Because the ratio was right-skewed, it was natural-log transformed for regression analysis. Regression coefficients and corresponding 95% CIs were exponentiated and are therefore presented as adjusted ratios. An adjusted ratio below 1 indicates a lower molar 25(OH)D/1,25(OH)_2_D ratio relative to the nonpregnant reference group. In the primary analysis, all observed total 1,25(OH)_2_D concentrations were retained irrespective of pregnancy status. As a sensitivity analysis, total 1,25(OH)_2_D concentrations >480 pmol/L were excluded in both pregnant and nonpregnant women, and the molar ratios and age-adjusted models were recalculated using the same procedures.

### **Additional sensitivity analyses**

Sensitivity analyses were performed to assess the influence of analyte-specific high-value handling. For total 25(OH)D, the primary analysis, in which concentrations >375 nmol/L were treated as missing, was compared with an analysis in which all observed values were retained. For total 1,25(OH)_2_D, all observed concentrations were retained in the primary analysis irrespective of pregnancy status; as a sensitivity analysis, concentrations >480 pmol/L were treated as missing in both pregnant and nonpregnant women. For free 25(OH)D, all observed concentrations were retained in the primary analysis, and a sensitivity analysis was performed in which concentrations above the 99th percentile of the combined study population were treated as missing. High-value handling was applied separately to each analyte and did not affect the availability of the other vitamin D measurements from the same participant.

### **Statistical methods for meta-analysis**

The ratio of means (ROM) was calculated on the logarithmic scale. For studies reporting arithmetic means, standard errors were estimated using the delta method, whereas for studies reporting geometric means, standard errors were derived from reported confidence intervals. Random-effects models were fitted using restricted maximum likelihood with Knapp-Hartung adjustment. Results were back-transformed and presented as ROM (pregnant/control) with 95% confidence intervals.

Statistical heterogeneity was assessed using the I² statistic and interpreted according to Higgins et al. Reporting bias was assessed visually using funnel plots and formally using Egger’s test. To assess whether the pooled estimates were dependent on inclusion of the current cohort, sensitivity meta-analyses were performed after excluding the current study. Additional robustness analyses included leave-one-out analyses and influence diagnostics, including studentized residuals, DFFITS, Cook’s distance, covariance ratio, heterogeneity after omitting each study, leverage, and study weight.

# Supplementary Figure S1. Study screening flow diagram.

# Supplementary Figure S2. Association between serum 25-hydroxyvitamin D and 1,25-dihydroxyvitamin D concentrations by pregnancy status.

Scatterplots with fitted linear regression lines showing positive associations between serum 25(OH)D and serum 1,25(OH)_2_D concentrations in both groups. Shaded areas represent 95% CIs. The slope of the association was steeper in pregnant women (*β* = 1.35, *P* < 0.001) than in non-pregnant women (*β* = 0.75, *P* < 0.001).

# Supplementary Figure S3. Funnel plot for the meta-analysis of studies assessing circulating 1,25-dihydroxyvitamin D concentrations in pregnant versus nonpregnant control participants.

This funnel plot shows the standard error and ratio of means (Preg/Ctrl) for each study included in the meta-analysis. The dashed vertical line indicates the pooled effect estimate. Egger’s test for funnel plot asymmetry was not statistically significant (*t* = -0.92, *df* = 7, *P* = 0.386).

# Supplementary Figure S4. Influence diagnostics for the primary meta-analysis of 1,25-dihydroxyvitamin D.

The panels show study-level influence statistics after omitting one study at a time, including studentized residuals (rstudent), DFFITS, Cook’s distance (cook.d), covariance ratio (cov.r), heterogeneity after deleting each study (tau2.del), Q statistic after deleting each study (QE.del), leverage (hat), and study weight. Red markers indicate the study that appeared relatively more influential for the corresponding diagnostic metric.

# Supplementary Table S1. Total 1,25-dihydroxyvitamin D and Pregnancy: Study Classification

| Citation First Author/year | Race | Design & population | Gestational window | Assay | Key findings |
| --- | --- | --- | --- | --- | --- |
| Kumar R et al., 1979 [4] | Predominantly Caucasian | Cross-sectional study including: normal pregnant women (*N* = 31)postpartum lactating women (*N* = 6), age-matched nonpregnant female controls(*N* = 10) | First, second, and third trimester; postpartum lactation also assessed | RRA | Plasma 1,25(OH)_2_D concentrations were significantly elevated in pregnant women compared with nonpregnant controls.  Levels remained elevated during lactation. |
| Steichen JJ et al., 1980 [ S1] | U.S. cohort | Observational study of term pregnant women (*N* = 19) with maternal, cord, and neonatal samples | Term pregnancy | RIA | Maternal serum 1,25(OH)_2_D concentrations at term were elevated above normal adult reference values. |
| Bouillon R et al., 1981 [22] | Belgium/European | Cross-sectional study including nonpregnant women (*N* = 58) and pregnant women sampled (*N* = 40) at multiple gestational ages (18, 32, 35, and 40 weeks); cord blood was also collected at 35 and 40 weeks. | Mid-to-late pregnancy | RRA | Maternal serum 1,25(OH)_2_D concentrations were approximately twofold higher during pregnancy compared with nonpregnant women. |
| Whitehead M et al., 1981 [S2] | UK (white population) | Cross-sectional study of healthy pregnant women and non-pregnant controls | 10–12, 20–22, 30–32, 36–40 weeks | RRA | Total 1,25(OH)_2_D concentrations increased significantly throughout pregnancy |
| Seino Y et al., 1982 [S3] | Japan | Cross-sectional study of term pregnant women (*N* = 30) with paired maternal, cord, and infant serum; adult controls (*N* = 20) | Term pregnancy (delivery) | Competitive protein-binding assays | Maternal and cord 1,25(OH)_2_D concentrations were significantly higher than adult levels |
| Reddy GS et al., 1983 [35] | Healthy adult women (United States) | Longitudinal and cross-sectional study of healthy pregnant women (*N* = 25) carrying a single fetus and pregnant women (*N* = 27) carrying twin fetuses. | First, second, third trimester; delivery; postpartum | RRA | 1,25(OH)_2_D concentrations rose from early to late pregnancy, reached highest levels at delivery, and decreased postpartum. |
| Markestad T et al., 1983 [30] | Caucasian | Comparative study including nonpregnant women (*N* = 17) and pregnant women at delivery (*N* = 22) | At delivery and 16 days postpartum | RRA | 1,25(OH)_2_D was significantly elevated during pregnancy compared with nonpregnant women and declined rapidly postpartum. |
| Bikle DD et al., 1984 [6] | US cohort | Cross-sectional study including normal subjects (*N* = 24), pregnant subjects (*N* = 17), and subjects with liver disease (*N* = 25) | Third trimester | RRA | Total 1,25(OH)_2_D was significantly increased in pregnant women compared with normals. |
| Okonofua F et al., 1987 [S4] | Asian & Caucasian | Cross-sectional observational study of pregnant women (*N* = 98) and neonates | 10–40 weeks + labour | RRA | 1,25(OH)_2_D concentrations at term were higher than non-pregnant reference values. |
| Nishioka T et al., 1988 [S5] | Japanese | Observational study, healthy pregnant women (*N* = 28) and neonates | Term delivery | Competitive binding assay | Maternal 1,25(OH)_2_D elevated; cord levels lower; neonatal levels rise rapidly after birth |
| Hoogenboezem T et al., 1989 [S6] | Caucasian | Longitudinal study of pregnant women (*N* = 39) and infants | Late pregnancy (≈6 week before delivery) and delivery | RRA | Maternal total 1,25(OH)_2_D about 2× adult reference during pregnancy; levels decline after delivery |
| Wilson SG et al., 1990 [5] | Caucasian | Prospective longitudinal study including healthy pregnant women (*N* = 35) followed through late pregnancy and lactation, and nonpregnant female controls (*N* = 26). | Late pregnancy (34–40 weeks), and postpartum (1–32 weeks). | Cytoreceptor/ Competitive protein-binding assay | both total and free 1,25(OH)_2_D rise during pregnancy despite increased DBP concentrations, indicating genuine upregulation of vitamin D activation rather than altered binding alone. |
| Seki K et al., 1991 [S7] | Japanese | Longitudinal study of healthy pregnant women (*N* = 20) followed across pregnancy. | Longitudinal study of healthy pregnant women followed across pregnancy. | RIA | Serum 1,25(OH)_2_D concentrations increased progressively across pregnancy, with significantly higher levels in late gestation compared with early pregnancy. |
| Cross NA et al., 1995 [S8] | Predominantly Caucasian | Longitudinal study of healthy women (*N* = 10) followed during pregnancy, lactation, and postweaning. | Serial measurements across pregnancy, during lactation, and after weaning. | RRA | Serum 1,25(OH)_2_D concentrations increased during pregnancy, declined during lactation, and returned toward baseline levels after weaning. |
| Seely EW et al., 1997 [S9] | White women | Healthy pregnant women (*N* = 23) | 2nd & 3rd trimesters vs postpartum | RIA | 1,25(OH)_2_D levels were equivalent in the second and third trimesters but were twofold higher than postpartum values |
| Sanchez PA et al., 1997 [31] | African (Nigerian population) | Cross-sectional study of pregnant adolescents (*N* = 30) | First, second, and third trimester. | RRA | 1,25(OH)_2_D concentrations were significantly higher in the second and third trimesters compared with nonpregnant controls. |
| Ardawi MS et al., 1997 [S10] | Saudi Arabia | Prospective longitudinal study of healthy pregnant women (*N* = 40) | 8–14 weeks, 17–27 weeks, 29–35 weeks, term, postpartum | RRA | Total 1,25(OH)_2_D increased markedly during pregnancy, |
| van Hoof HJ et al., 2001 [32] |  | Cross-sectional study including pregnant women (*N* = 10) and healthy controls | Third trimester | RRA | Pregnant women had significantly higher total 1,25(OH)_2_D |
| Uemura H et al., 2002 [S11] | Japanese women | Longitudinal study of healthy women (*N* = 14) followed during pregnancy and lactation | Serial measurements across pregnancy,  Early postpartum  Lactation | RIA | Serum 1,25(OH)_2_D increased during pregnancy, decreased immediately postpartum, and partially rebounded at 1 month. |
| Young BE et al., 2012 [S12] | Multiracial cohort (United States; pregnant adolescents) | Prospective longitudinal study of healthy pregnant adolescents (*N* = 171). | Mid-gestation and at delivery (with cord blood samples collected at birth). | Thymus Receptor Binding Assay | Serum 1,25(OH)_2_D concentrations were elevated at mid-gestation, declined modestly at delivery , and were substantially lower in cord blood |
| O’Brien KO et al., 2012 [S13] | African American / White | Prospective longitudinal study of healthy pregnant women (*N* = 46). | Early pregnancy (~12 weeks), late pregnancy (~34–36 weeks), and early postpartum. | RIA | Serum 1,25(OH)_2_D concentrations increased significantly from early to late pregnancy. |
| Møller UK et al., 2013 [28] | Predominantly Caucasian | Controlled longitudinal cohort study including healthy pregnant women (*N* = 153) and nonpregnant controls (*N* = 52). | Pregnancy (multiple time points across gestation) and postpartum | RIA | Contrary to the expected rise in active vitamin D during pregnancy, baseline 1,25(OH)_2_D levels were significantly lower in pregnant women compared with non-pregnant controls. |
| Jones KS et al., 2016 [33] | Rural Gambian women (West Africa) | healthy pregnant women (*N* = 22), nonpregnant nonlactating controls (*N* = 23) | Cross-sectional comparison | RIA | Tota 1,25(OH)_2_D concentrations were significantly higher in pregnancy compared with non-pregnant women. |
| Park H et al., 2016 [34] | Mostly Caucasian women | Healthy pregnant women (*N* = 26) and healthy Non-pregnant women (*N* = 21) | First trimester | RIA | Circulating total 1,25(OH)_2_D concentrations were significantly higher in pregnant women than in non-pregnant controls at both baseline and study-end |
| Figueiredo ACC et al., 2018 [39] | Brazilian healthy pregnant women | Prospective longitudinal cohort study including healthy pregnant women (*N* = 229) | 5–13, 20–26, 30–36 weeks | LC-MS/MS | 1,25(OH)_2_D increased significantly with gestational age |
| Gustafsson MK et al., 2018 [38] | Norway | Prospective longitudinal cohort of healthy pregnant women (*N* = 855) | 18–22 and 32–36 weeks | Immunoassay | 1,25(OH)_2_D increased during pregnancy. |
| Best CM et al., 2019 [S14] | Multiracial cohort (pregnant adolescents, United States) | Prospective longitudinal study including pregnant adolescents (*N* = 58) with serial measurements during pregnancy; | Entry visit (early pregnancy), mid-pregnancy, and at delivery. | LC–MS/MS | 1,25(OH)_2_D concentrations were elevated in early pregnancy, peaked at mid-gestation, and declined toward delivery. |
| Perreault M et al., 2020 [S15] | Canada | Prospective cohort of healthy pregnant women (*N* = 187) | 12–17 weeks → 36–38 weeks | LC-MS/MS | 1,25(OH)_2_D increased from early to late pregnancy; adequate vitamin D status maintained with supplementation and summer exposure |
| Tsuprykov O et al., 2021 [S16] | Germany | Cross-sectional study including healthy pregnant women (*N* = 427) across different gestational stages. | First, second, and third trimester | Automated Immunoassay (Chemiluminescence-Based Method). | Serum 1,25(OH)_2_D concentrations increased with advancing gestational age. |
| Best CM et al., 2022 [40] | USA (likely mixed ethnicity) | Controlled kinetic study including nonpregnant women (*N* = 6) and healthy pregnant women (*N* = 4) receiving a single oral dose of labeled vitamin D₃. | Second–Third trimester | LC–MS/MS | Pregnant women exhibited higher baseline 1,25(OH)_2_D concentrations compared with nonpregnant women. |

Abbreviations: Sample size (N), Radioreceptor assay (RRA), Radioimmunoassay (RIA).

# Supplementary Table S2. Free 25-hydroxyvitamin D and Pregnancy: Study Classification

| Citation  First Author/year | Race | Design & population | Gestational window | Vitamin D metrics | Key Findings |
| --- | --- | --- | --- | --- | --- |
| Bouillon R et al., 1977 [S17] | Belgium (likely Caucasian) | Paired maternal–cord serum study (*N* = 30) | Delivery (~40 week) | Calculated free 25(OH)D | Maternal total 25(OH)D higher than cord, but free 25(OH)D slightly higher in cord serum, |
| Bouillon R et al., 1981 [29] | Mostly Caucasian women | Cross-sectional study including nonpregnant women, pregnant women at different gestational stages, cord blood, and postpartum women | Mid-to-late pregnancy | Free 25(OH)D index calculated from total 25(OH)D and vitamin D–binding protein measurements | Calculated free 25(OH)D concentrations (free 25-OHD index) were reduced during pregnancy compared with nonpregnant women |
| Zhang JY et al*.*, 2014 [S18] | Caucasian (Irish) | Prospective longitudinal study  of healthy pregnant women (*N* = 30) | Longitudinally at 15, 20, 24, 28, 32, 36, 40 weeks and 2 months postpartum | Free 25(OH)D was calculated from total 25(OH)D, DBP and albumin; | Calculated free concentrations decreased as gestation progressed, with the lowest values observed in the third trimester. |
| Schwartz JB et al*.*, 2014 [41] | Mostly Caucasians | Cross-sectional clinical population study including pregnant women (*N* = 20), healthy controls (*N* = 111), and cirrhosis patient (*N* = 24) | Second and Third trimesters | Direct measurement using free ELISA kit by Future Diagnosticsa and assessment by calculation based on DBP levels obtained by monoclonal DBP ELISA assay | Directly measured free was significantly lower. The study also highlighted that calculated free values often differ from measured values in pregnancy due to changes in binding protein affinity. |
| Jones KS et al., 2016 [33] | The Gambia | Stable-isotope kinetic study including pregnant (*N* = 22), lactating (*N* = 14), and non-pregnant women (*N* = 10) | Third trimester | Free 25(OH)D calculated from total 25(OH)D, vitamin D–binding protein, and albumin concentrations (not directly measured). | Calculated free 25(OH)D concentrations did not differ significantly between pregnant and nonpregnant women |
| Park H et al*.*, 2016 [34] | Mostly Caucasian women | Cross-sectional comparison of healthy pregnant women (*N* = 26) and healthy non-pregnant women (*N* = 21) | First trimester | Free 25(OH)D calculated from total 25(OH)D, vitamin D-binding protein, and albumin concentrations (not directly measured). | Calculated free 25(OH)D concentrations (free 25-OHD index) were reduced during pregnancy compared with nonpregnant women. |
| Gustafsson MK et al., 2018 [38] | Mostly Caucasian (Norwegian) | Prospective longitudinal cohort of healthy pregnant women (*N* = 855) | Early, mid-, and late pregnancy (longitudinal follow-up across gestation) | Free 25(OH)D calculated from total 25(OH)D, vitamin D–binding protein, and albumin concentrations | Calculated free index decreased significantly from the second to the third trimester . |
| Tsuprykov O et al.*,* 2018 & 2019 [9, 10] | Germany | Cross-sectional observational study of healthy pregnant women (*N* = 368) | First, second, and third trimesters | Measured  free 25(OH)D  (ELISA), and calculated free 25(OH)D | Free 25(OH)D levels (regardless of the determination approach) were found to be significantly decreased in the 3rd trimester compared to the 1st trimester |
| Schwartz JB et al., 2018 [S19] | Mostly Caucasians | Cross-sectional observational study including multiple clinical populations and pregnant women （*N* = 20） | Not specified | Measured free 25(OH)D | Free 25(OH)D varied widely across populations and correlated with total 25(OH)D and DBP |
| Pereira JN et al*.*, 2020 [S20] | Brazil | Cross-sectional observational study of pregnant women (*N* = 123) | 27–41 weeks | Calculated free 25(OH)D | Maternal organism is able to maintain free 25(OH)D3 fraction independent on gestational age. |
| Fernando M et al., 2020 & 2020 [S21,S22] | Predominantly Caucasian women (Australian population) | Retrospective cohort study of pregnant women (*N* = 304) | Early pregnancy | Free 25(OH)D calculated from total 25(OH)D, vitamin D–binding protein, and albumin concentrations (not directly measured). | Higher maternal age was correlated with lower VDBP and higher free and bioavailable 25(OH)D concentrations  Higher maternal total and free 25(OH)D concentrations were associated with higher neonatal birthweight |
| Best C.M. et al., 2019 [S14] | Multiracial cohort (predominantly African American and Hispanic adolescents) | Pregnant adolescents with repeated measurements across gestation | Early, mid-, and late pregnancy (longitudinal follow-up) | ELISA | Free 25(OH)D did not change significantly between entry and delivery |
| Xia J et al.,2019 [S23] | Multiracial cohort | Prospective longitudinal cohort study of pregnant women and gestational diabetes assessment | Early to mid-pregnancy (multiple time points) | Calculated free and bioavailable 25(OH)D | In both GDM and control groups, median levels of , , total , and free increased across gestation. |
| Tsuprykov et al*.*, 2021 [S16] | Germany | Cross-sectional observational study of healthy pregnant women (*N* = 427) | 1st–3rd trimester | Calculated free 25(OH)D | Free and bioavailable 25(OH)D correlated better with gestational age and metabolic biomarkers, while total 1,25(OH)₂D showed stronger associations with endocrine parameters |
| George JA et al*.*, 2021 [S24] | South Africa | Prospective longitudinal cohort study of pregnant women (*N* = 400) | Early pregnancy, late pregnancy, postpartum | Calculated free 25(OH)D | Free and bioavailable vitamin D dropped, but these were not statistically significant |
| Best CM et al*.*, 2022 [40] | USA | Stable-isotope pharmacokinetic pilot study including pregnant (*N* = 4) and non-pregnant women (*N* = 6) | Not specified | ELISA | Free 25(OH)D lower in pregnancy |
| Le J et al*.*, 2023 [S25] | China | Cross-sectional study comparing singleton and twin pregnancies | Not specified | Calculated free and bioavailable 25(OH)D | Free 25(OH)D concentrations were lower in twin pregnancies compared with singleton pregnancies, indicating reduced bioavailable vitamin D status in twin gestations |
| McWhorter CA et al*.*, 2023 [S26] | USA | Post-hoc analysis of a randomized controlled trial cohort of pregnant women (*N* = 297) | Early pregnancy and 5–7 months gestation | ELISA | Lower free 25(OH)D/PTH ratio was associated with the development of gestational diabetes during pregnancy |
| Stunes AK et al*.*, 2024 [S27] | Norway | Post-hoc analysis of a randomized controlled trial cohort of pregnant women (*N* = 119) | 2nd and 3rd trimester | Calculated free and bioavailable 25(OH)D | Maternal free 25(OH)D concentrations during pregnancy were not associated with offspring bone mineral content at 9 years |
| Fakhoury HMA et al., 2024 [S28] | Lebanon (Mediterranean) | Cross-sectional mother–child cohort study of pregnant women (*N* = 70) | Delivery | Calculated free and bioavailable 25(OH)D | Both maternal free and total 25(OH)D forms were independently associated with all neonatal vitamin D forms |
| Mercer S et al*.*, 2025 [S29] | Multiracial U.S. cohort | Prospective longitudinal cohort study of pregnant women (*N* = 50) | Early to late pregnancy | ELISA | Free 25(OH)D exhibited a U-shaped pattern during pregnancy, decreasing in mid-gestation and increasing again later in pregnancy |
| Tian Z et al*.*, 2025 [S30] | China | Cross-sectional mother–infant cohort study (*N* = 160) | Delivery | ELISA | Free 25(OH)D in maternal and umbilical blood showed stronger correlations with fetal growth parameters than total 25(OH)D |

Abbreviations: Sample size ( N ), Enzyme-Linked Immunosorbent Assay ( ELISA )

# **Supplementary Table S3. Sensitivity analysis of high-value handling for total 25-hydroxyvitamin D.**

| **Group** | **Primary analysis** | | | **All values retained** | | |
| --- | --- | --- | --- | --- | --- | --- |
|  | **n** | **Age-adjusted β (95% CI), pmol/L** | **P** | **n** | **Age-adjusted β (95% CI), pmol/L** | **P** |
| Nonpregnant women | 101,104 | 0 (Reference) | — | 101,249 | 0 (Reference) | — |
| Pregnant women | 15,938 | 22.07 (21.50–22.63) | <0.001 | 15,947 | 21.57 (20.90–22.24) | <0.001 |
| 1st trimester (Early) | 7,871 | 10.60 (9.83–11.37) | <0.001 | 7,879 | 10.35 (9.43–11.26) | <0.001 |
| 2nd trimester (Mid) | 4,595 | 30.08 (29.09–31.07) | <0.001 | 4,596 | 29.39 (28.21–30.57) | <0.001 |
| 3rd trimester (Late) | 3,472 | 37.40 (36.27–38.53) | <0.001 | 3,472 | 36.64 (35.29–37.99) | <0.001 |

Values are age-adjusted β coefficients with 95% confidence intervals. High-value handling was analyte-specific: total 25(OH)D concentrations >375 nmol/L were treated as missing in the primary analysis and retained in the sensitivity analysis. Nonpregnant women served as the reference group.

# **Supplementary Table S4. Sensitivity analysis excluding total 1,25-dihydroxyvitamin D concentrations >480 pmol/L in both groups.**

| **Group** | **Primary analysis** | | | **>480 pmol/L excluded in both groups** | | |
| --- | --- | --- | --- | --- | --- | --- |
|  | **n** | **Age-adjusted β (95% CI), pmol/L** | **P** | **n** | **Age-adjusted β (95% CI), pmol/L** | **P** |
| Nonpregnant women | 5,952 | 0 (Reference) | — | 5,942 | 0 (Reference) | — |
| Pregnant women | 183 | 125.20 (115.39–135.02) | <0.001 | 178 | 117.77 (108.32–127.22) | <0.001 |
| 1st trimester (Early) | 49 | 67.37 (48.69–86.04) | <0.001 | 49 | 68.09 (50.35–85.84) | <0.001 |
| 2nd trimester (Mid) | 96 | 137.99 (124.60–151.39) | <0.001 | 93 | 129.37 (116.44–142.30) | <0.001 |
| 3rd trimester (Late) | 38 | 167.42 (146.24–188.61) | <0.001 | 36 | 155.39 (134.71–176.07) | <0.001 |

Note: Values are age-adjusted β coefficients with 95% confidence intervals. In the primary analysis, all observed total 1,25(OH)₂D concentrations were retained irrespective of pregnancy status. As a sensitivity analysis, concentrations >480 pmol/L were treated as missing in both pregnant and nonpregnant women. Nonpregnant women served as the reference group in all models. n represents the number of participants included in each corresponding group under each high-value handling strategy.

# **Supplementary Table S5. Sensitivity analysis of extreme-value handling for free 25-hydroxyvitamin D.**

| **Group** | **Primary analysis** | | | **Sensitivity analysis** | | |
| --- | --- | --- | --- | --- | --- | --- |
|  | **n** | **Age-adjusted β (95% CI), pmol/L** | **P** | **n** | **Age-adjusted β (95% CI), pmol/L** | **P** |
| Nonpregnant women | 304 | 0 (Reference) | — | 300 | 0 (Reference) | — |
| Pregnant women | 60 | −2.63 (−8.99–3.74) | 0.417 | 60 | −1.32 (−5.79–3.16) | 0.564 |
| 1st trimester (Early) | 34 | −1.68 (−9.84–6.48) | 0.686 | 34 | −0.53 (−6.27–5.21) | 0.856 |
| 2nd trimester (Mid) | 15 | −4.08 (−15.91–7.75) | 0.498 | 15 | −2.77 (−11.09–5.55) | 0.513 |
| 3rd trimester (Late) | 11 | −3.54 (−17.21–10.14) | 0.611 | 11 | −1.73 (−11.35–7.88) | 0.723 |

Values are age-adjusted β coefficients with 95% confidence intervals. In the primary analysis, all observed free 25(OH)D concentrations were retained. In the sensitivity analysis, free 25(OH)D concentrations above the 99th percentile of the combined study population were treated as missing. Extreme-value handling was applied to free 25(OH)D only and did not affect total 25(OH)D or total 1,25(OH)₂D analyses. Nonpregnant women served as the reference group.

# **Supplementary Table S6 . Main and repeated equal-size random subsampling analyses of pregnancy-associated differences in circulating vitamin D metabolites**

| Vitamin D metabolite | Main analysis | | Repeated equal-size random subsampling | |
| --- | --- | --- | --- | --- |
|  | n  (NP/P) | β (95% CI) | n per iteration (NP/P) | Mean β (2.5th–97.5th percentile) |
| Total 25(OH)D | 101,104 / 15,938 | 22.07 (21.50, 22.63) | 15,938 / 15,938 | 22.36 (21.86, 22.88) |
| Total 1,25(OH)_2_D | 5,952 / 183 | 125.20 (115.39, 135.02) | 183 / 183 | 126.43 (117.75, 134.84) |
| Free 25(OH)D | 304 / 60 | −2.63 (−8.99, 3.74) | 60 / 60 | −2.64 (−7.91, 1.62) |

Abbreviations: CI, confidence interval; NP, nonpregnant; P, pregnant.
β coefficients represent the estimated difference in circulating vitamin D metabolite concentrations associated with pregnancy status, with nonpregnant women as the reference group. Main analyses were performed using the full analyte-specific cohorts and adjusted for age. For the repeated equal-size random subsampling analysis, all pregnant participants were retained, and an equal number of nonpregnant women were randomly sampled from the corresponding analyte-specific cohort in each iteration. Age-adjusted linear regression was performed in each of 1,000 iterations. Values are presented as the mean β and the empirical 2.5th–97.5th percentiles of β estimates across iterations. The percentile range describes the distribution of estimates across repeated random subsamples and does not represent a model-based 95% CI.

# **Supplementary Table S7. Comparison of available characteristics between participants with and without specialized vitamin D metabolite measurements**

| **Population** | **Characteristic** | **Total 1,25(OH)_2_D measurement** | | | **Free 25(OH)D measurement** | | |
| --- | --- | --- | --- | --- | --- | --- | --- |
|  |  | **Measured** | **Not measured** | **Absolute SMD** | **Measured** | **Not measured** | **Absolute SMD** |
| **Pregnant** | n | 183 | 15920 | — | 60 | 15,957 | — |
|  | Age, years | 32 (30–35) | 33 (30–36) | 0.085 | 33 (29–35) | 33 (30–36) | 0.149 |
|  | Gestational week | 24 (13–27) | 14 (8–26) | 0.375 | 11.43 (8–24.25) | 14 (8–26) | 0.098 |
|  | Total 25(OH)D, nmol/L | 78.8 (55.2–95.0), n=162 | 80.0 (60.0–100.0), n=15,861 | 0.129 | 105 (78.8–111), n=11 | 80.0 (60.0–100.0), n=15,927 | 0.415 |
| **Nonpregnant** | n | 5,952 | 95,502 | — | 304 | 101,150 | — |
|  | Age, years | 35 (28–40) | 35 (29–40) | 0.027 | 38 (31–42) | 35 (29–40) | 0.202 |
|  | Total 25(OH)D, nmol/L | 53.0 (32.5–75.0), n=5,857 | 57.5 (37.5–77.5), n=95,247 | 0.108 | 70.0 (55.0–107.5), n=181 | 57.5 (37.5–77.5), n=100,923 | 0.605 |

**Note:** Values are median (IQR) unless otherwise indicated. SMD, standardized mean difference. Measurement status was defined according to the availability of the original assay result before analyte-specific extreme-value handling. Absolute SMDs based on means and pooled standard deviations were used to quantify differences in available continuous characteristics between participants with and without the respective specialized metabolite measurement. Total 25(OH)D comparisons were restricted to participants with available total 25(OH)D measurements, with the corresponding sample sizes shown in the table. These comparisons assess similarity in observed characteristics only and do not establish representativeness with respect to unmeasured clinical characteristics or indications for specialized testing.

# **Supplementary Table S8. Primary and sensitivity analyses of the molar ratio of total 25(OH)D to total 1,25(OH)_2_D according to pregnancy status and gestational trimester**

| **Group** | **Primary analysis, n** | **25(OH)D/1,25(OH)_2_D molar ratio, median (IQR)** | **Primary analysis, adjusted ratio (95% CI)** | **Sensitivity analysis, n** | **Sensitivity analysis, adjusted ratio (95% CI)** |
| --- | --- | --- | --- | --- | --- |
| Nonpregnant | 5,857 | 392.9 (260.4–575.6) | Reference | 5,849 | Reference |
| Pregnant overall | 162 | 289.8 (210.9–394.5) | 0.754 (0.684–0.831) | 157 | 0.753 (0.682–0.831) |
| T1 | 40 | 256.1 (185.6–430.1) | 0.693 (0.572–0.842) | 40 | 0.693 (0.571–0.841) |
| T2 | 87 | 304.9 (257.6–396.8) | 0.806 (0.707–0.920) | 84 | 0.807 (0.706–0.923) |
| T3 | 35 | 258.2 (199.4–365.1) | 0.703 (0.572–0.865) | 33 | 0.698 (0.565–0.864) |

**Note:** Values for the molar ratio are presented as median (IQR). The molar ratio was calculated after converting total 25(OH)D from nmol/L to pmol/L. Adjusted ratios were obtained by exponentiating coefficients from age-adjusted linear regression models using the natural logarithm of the 25(OH)D/1,25(OH)₂D ratio as the dependent variable, with nonpregnant women as the reference group. In the primary analysis, all observed total 1,25(OH)₂D concentrations were retained irrespective of pregnancy status. In the sensitivity analysis, total 1,25(OH)₂D concentrations >480 pmol/L were treated as missing in both pregnant and nonpregnant women. T1 was defined as ≤13 weeks + 6 days, T2 as 14 weeks to 27 weeks + 6 days, and T3 as ≥28 weeks until delivery.

# **Supplementary Table S9. Available participant characteristics and analyte-specific vitamin D measurements according to pregnancy status**

| **Characteristic** | **Nonpregnant women** | **Pregnant women** |
| --- | --- | --- |
| Overall study population, n | 101,454 | 16,017 |
| Age, years, median (IQR) | 35.00 (29.00–40.00) | 33.00 (30.00–36.00) |
| Gestational age, weeks, median (IQR) | - | 14.00 (8.00–26.00) |
| **Total 25(OH)D** |  |  |
| n | 101,104 | 15,938 |
| Median (IQR), nmol/L | 57.50 (37.50–77.50) | 80.00 (60.00–100.00) |
| **Total 1,25(OH_2_D** |  |  |
| n | 5,952 | 183 |
| Median (IQR), pmol/L | 126.72 (96.24–168.00) | 259.20 (202.92–312.00) |
| **Free 25(OH)D** |  |  |
| n | 304 | 60 |
| Median (IQR), pmol/L | 18.18 (13.05–30.00) | 19.62 (15.09–27.75) |

**Note:** Values are presented as n or median (IQR), as appropriate. Vitamin D metabolites were measured in separate analyte-specific subcohorts because testing was performed selectively in routine clinical practice. No participant had valid measurements of all three vitamin D biomarkers simultaneously; therefore, the table does not represent a common complete-case cohort. Gestational age is applicable only to pregnant women.

# **Supplementary Table S10. Primary and log-transformed sensitivity analyses for the main age-adjusted regression models.**

| Model | Primary analysis: Estimate (95% CI) | *P* value | Log-transformed sensitivity analysis: Estimate (95% CI) | *P* value | Overall conclusion unchanged |
| --- | --- | --- | --- | --- | --- |
| Total 25(OH)D | 22.07 (21.50, 22.63) | <0.001 | 0.376 (0.366, 0.386) | <0.001 | Yes |
| Total 1,25(OH)_2_D | 125.20 (115.39, 135.02) | <0.001 | 0.673 (0.601, 0.745) | <0.001 | Yes |
| Free 25(OH)D | -2.63 (-8.99, 3.74) | 0.417 | 0.043 (-0.130, 0.216) | 0.625 | Yes |

Primary analyses used age-adjusted linear regression models with pregnancy status as the exposure and nonpregnant women as the reference group. Sensitivity analyses used the same model specification with log-transformed outcomes. Primary estimates represent adjusted mean differences on the original scale, whereas log-transformed estimates represent regression coefficients on the logarithmic scale. Overall conclusions were considered unchanged when the direction and statistical interpretation of the associations were consistent between the primary and sensitivity analyses.

# **Supplementary Table S11. Regression diagnostics for the log-transformed age-adjusted models**

| Outcome | Breusch–Pagan P value | Maximum Cook’s distance | Maximum leverage | Maximum absolute studentized residual |
| --- | --- | --- | --- | --- |
| Total 25(OH)D | <0.001 | 0.00035 | 0.00035 | 4.679 |
| Total 1,25(OH)_2_D | <0.001 | 0.01984 | 0.00590 | 5.399 |
| Free 25(OH)D | 0.002 | 0.03409 | 0.02283 | 3.812 |

The Breusch–Pagan test was used to assess heteroscedasticity. Cook’s distance, leverage, and absolute studentized residuals were used to assess potentially influential observations. These diagnostic measures were interpreted collectively and were not used as automatic exclusion criteria.

# **Supplementary Table S12. Primary and sensitivity meta-analyses of vitamin D metabolites in pregnant versus nonpregnant women**

| **Metabolite** | **Analysis** | **Studies, n** | **Pooled ROM (95% CI)** | **Interpretation** |
| --- | --- | --- | --- | --- |
| Total 1,25(OH)₂D | Primary meta-analysis | 9 | 1.88 (1.67–2.12) | Higher in pregnancy |
|  | Excluding current cohort | 8 | 1.87 (1.62–2.16) | Essentially unchanged |
| Free 25(OH)D | Primary meta-analysis | 3 | 0.78 (0.63–0.97) | Lower in pregnancy |
|  | Excluding current cohort | 2 | 0.74 (0.41–1.33) | Similar direction; CI crossed 1 |

**Note.** ROM indicates ratio of means (pregnant/nonpregnant women). Random-effects models used restricted maximum likelihood with Knapp–Hartung adjustment. For total 1,25(OH)₂D, I² was 76.68% in the primary analysis and 74.14% after exclusion of the current cohort. For the free 25(OH)D sensitivity analysis, I² was 0%; with only two remaining studies, heterogeneity estimates should be interpreted cautiously.

# **Supplementary Table S13. JBI critical appraisal of analytical cross-sectional studies included in the meta-analysis.**

| Study | Q1 | Q2 | Q3 | Q4 | Q5 | Q6 | Q7 | Q8 |
| --- | --- | --- | --- | --- | --- | --- | --- | --- |
| Kumar 1979 | U | Y | Y | Y | N | N | Y | Y |
| Bouillon 1981 | Y | Y | Y | Y | N | N | Y | Y |
| Markestad 1983 | Y | Y | Y | Y | N | N | Y | Y |
| Sanchez 1997 | Y | Y | Y | Y | Y | N | Y | Y |
| van Hoof 2001 | Y | Y | Y | Y | Y | N | Y | Y |
| Park 2016 | Y | Y | Y | Y | Y | Y | Y | Y |
| Zhang 2026 | Y | Y | Y | Y | Y | Y | Y | Y |

**Abbreviations:** Y, yes; N, no; U, unclear; NA, not applicable.
**Q1:** Were the criteria for inclusion in the sample clearly defined?
**Q2:** Were the study subjects and the setting described in detail?
**Q3:** Was the exposure measured in a valid and reliable way?
**Q4:** Were objective, standard criteria used for measurement of the condition?
**Q5:** Were confounding factors identified?
**Q6:** Were strategies to deal with confounding factors stated?
**Q7:** Were the outcomes measured in a valid and reliable way?
**Q8:** Was appropriate statistical analysis used?

# **Supplementary Table S14. JBI critical appraisal of cohort studies included in the meta-analysis.**

| Study | Q1 | Q2 | Q3 | Q4 | Q5 | Q6 | Q7 | Q8 | Q9 | Q10 | Q11 |
| --- | --- | --- | --- | --- | --- | --- | --- | --- | --- | --- | --- |
| Wilson 1990 | Y | Y | Y | N | N | Y | Y | Y | U | U | Y |
| Jones 2016 | Y | Y | Y | N | N | Y | Y | Y | U | U | Y |

**Abbreviations:** Y, yes; N, no; U, unclear; NA, not applicable.

**Q1:** Were the two groups similar and recruited from the same population?
**Q2:** Were the exposures measured similarly to assign people to both exposed and unexposed groups?
**Q3:** Was the exposure measured in a valid and reliable way?
**Q4:** Were confounding factors identified?
**Q5:** Were strategies to deal with confounding factors stated?
**Q6:** Were the groups/participants free of the outcome at the start of the study (or at the moment of exposure)?
**Q7:** Were the outcomes measured in a valid and reliable way?
**Q8:** Was the follow-up time reported and sufficient for outcomes to occur?
**Q9:** Was follow-up complete, and if not, were the reasons to loss to follow-up described and explored?
**Q10:** Were strategies to address incomplete follow-up utilized?
**Q11:** Was appropriate statistical analysis used?

# Supplementary References：

S1. Steichen, J.J.; Tsang, R.C.; Gratton, T.L.; Hamstra, A.; DeLuca, H.F. Vitamin D homeostasis in the perinatal period: 1,25-Dihydroxyvitamin D in maternal, cord, and neonatal blood. N. Engl. J. Med. 1980, 302, 315–319. doi:10.1056/NEJM198002073020603.
S2. Whitehead, M.; Lane, G.; Young, O.; Campbell, S.; Abeyasekera, G.; Hillyard, C.J.; MacIntyre, I.; Phang, K.G.; Stevenson, J.C. Interrelations of calcium-regulating hormones during normal pregnancy. Br. Med. J. (Clin. Res. Ed.) 1981, 283, 10–12. doi:10.1136/bmj.283.6283.10.
S3. Seino, Y.; Ishida, M.; Yamaoka, K.; Ishii, T.; Hiejima, T.; Ikehara, C.; Tanaka, Y.; Matsuda, S.; Shimotsuji, T.; Yabuuchi, H.; et al. Serum calcium-regulating hormones in the perinatal period. Calcif. Tissue Int. 1982, 34, 131–135. doi:10.1007/BF02411223.
S4. Okonofua, F.; Menon, R.K.; Houlder, S.; Thomas, M.; Robinson, D.; O’Brien, S.; Dandona, P. Calcium, vitamin D and parathyroid hormone relationships in pregnant Caucasian and Asian women and their neonates. Ann. Clin. Biochem. 1987, 24, 22–28. doi:10.1177/000456328702400103.
S5. Nishioka, T.; Yasuda, T.; Niimi, H.; Nakajima, H. Evidence that calcitonin plays a role in the postnatal increase of serum 1α,25-dihydroxyvitamin D. Eur. J. Pediatr. 1988, 147, 148–152. doi:10.1007/BF00442212.
S6. Hoogenboezem, T.; Degenhart, H.J.; de Muinck Keizer-Schrama, S.M.; Bouillon, R.; Grose, W.F.; Hackeng, W.H.; Visser, H.K. Vitamin D metabolism in breast-fed infants and their mothers. Pediatr. Res. 1989, 25, 623–628. doi:10.1203/00006450-198906000-00014.
S7. Seki, K.; Makimura, N.; Mitsui, C.; Hirata, J.; Nagata, I. Calcium-regulating hormones and osteocalcin levels during pregnancy: A longitudinal study. Am. J. Obstet. Gynecol. 1991, 164, 1248–1252. doi:10.1016/0002-9378(91)90694-M.
S8. Cross, N.A.; Hillman, L.S.; Allen, S.H.; Krause, G.F.; Vieira, N.E. Calcium homeostasis and bone metabolism during pregnancy, lactation, and postweaning: A longitudinal study. Am. J. Clin. Nutr. 1995, 61, 514–523. doi:10.1093/ajcn/61.3.514.
S9. Seely, E.W.; Brown, E.M.; DeMaggio, D.M.; Weldon, D.K.; Graves, S.W. A prospective study of calciotropic hormones in pregnancy and postpartum: Reciprocal changes in serum intact parathyroid hormone and 1,25-dihydroxyvitamin D. Am. J. Obstet. Gynecol. 1997, 176, 214–217. doi:10.1016/S0002-9378(97)80039-7.
S10. Ardawi, M.S.; Nasrat, H.A.; BA’Aqueel, H.S. Calcium-regulating hormones and parathyroid hormone-related peptide in normal human pregnancy and postpartum: A longitudinal study. Eur. J. Endocrinol. 1997, 137, 402–409. doi:10.1530/eje.0.1370402.
S11. Uemura, H.; Yasui, T.; Kiyokawa, M.; Kuwahara, A.; Ikawa, H.; Matsuzaki, T.; Maegawa, M.; Furumoto, H.; Irahara, M. Serum osteoprotegerin/osteoclastogenesis-inhibitory factor during pregnancy and lactation and the relationship with calcium-regulating hormones and bone turnover markers. J. Endocrinol. 2002, 174, 353–359. doi:10.1677/joe.0.1740353.
S12. Young, B.E.; McNanley, T.J.; Cooper, E.M.; McIntyre, A.W.; Witter, F.; Harris, Z.L.; O’Brien, K.O. Vitamin D insufficiency is prevalent and vitamin D is inversely associated with parathyroid hormone and calcitriol in pregnant adolescents. J. Bone Miner. Res. 2012, 27, 177–186. doi:10.1002/jbmr.526.
S13. O’Brien, K.O.; Donangelo, C.M.; Ritchie, L.D.; Gildengorin, G.; Abrams, S.; King, J.C. Serum 1,25-dihydroxyvitamin D and calcium intake affect rates of bone calcium deposition during pregnancy and the early postpartum period. Am. J. Clin. Nutr. 2012, 96, 64–72. doi:10.3945/ajcn.111.029231.
S14. Best, C.M.; Pressman, E.K.; Queenan, R.A.; Cooper, E.; O’Brien, K.O. Longitudinal changes in serum vitamin D-binding protein and free 25-hydroxyvitamin D in a multiracial cohort of pregnant adolescents. J. Steroid Biochem. Mol. Biol. 2019, 186, 79–88. doi:10.1016/j.jsbmb.2018.09.019.
S15. Perreault, M.; Atkinson, S.A.; Meyre, D.; Fusch, G.; BHIP Study Team. Summer season and recommended vitamin D intake support adequate vitamin D status throughout pregnancy in healthy Canadian women and their newborns. J. Nutr. 2020, 150, 739–746. doi:10.1093/jn/nxz276.
S16. Tsuprykov, O.; Elitok, S.; Buse, C.; Chu, C.; Krämer, B.K.; Hocher, B. Opposite correlation of 25-hydroxyvitamin D and 1,25-dihydroxyvitamin D metabolites with gestational age and bone and lipid biomarkers in pregnant women. Sci. Rep. 2021, 11, 1923. doi:10.1038/s41598-021-81452-9.
S17. Bouillon, R.; Van Baelen, H.; De Moor, P. 25-Hydroxyvitamin D and its binding protein in maternal and cord serum. J. Clin. Endocrinol. Metab. 1977, 45, 679–684. doi:10.1210/jcem-45-4-679.
S18. Zhang, J.Y.; Lucey, A.J.; Horgan, R.; Kenny, L.C.; Kiely, M. Impact of pregnancy on vitamin D status: A longitudinal study. Br. J. Nutr. 2014, 112, 1081–1087. doi:10.1017/S0007114514001883.
S19. Schwartz, J.B.; Gallagher, J.C.; Jorde, R.; Berg, V.; Walsh, J.; Eastell, R.; Evans, A.L.; Bowles, S.; Naylor, K.E.; Jones, K.S.; et al. Determination of free 25(OH)D concentrations and their relationships to total 25(OH)D in multiple clinical populations. J. Clin. Endocrinol. Metab. 2018, 103, 3278–3288. doi:10.1210/jc.2018-00295.
S20. Pereira, J.N.; Chactoura, J.; Nohra, F.; Diogenes, M.E.L.; Bezerra, F.F. Free and bioavailable fractions of vitamin D: Association with maternal characteristics in Brazilian pregnant women. J. Nutr. Metab. 2020, 2020, 1408659. doi:10.1155/2020/1408659.
S21. Fernando, M.; Ellery, S.J.; de Guingand, D.; Marquina, C.; Lim, S.; Harrison, C.L.; Teede, H.J.; Naderpoor, N.; Mousa, A. Early pregnancy vitamin D-binding protein is independently associated with the development of gestational diabetes: A retrospective cohort study. J. Clin. Med. 2020, 9, 2186. doi:10.3390/jcm9072186.
S22. Fernando, M.; Coster, T.G.; Ellery, S.J.; de Guingand, D.; Lim, S.; Harrison, C.L.; Teede, H.J.; Naderpoor, N.; Mousa, A. Relationships between total, free and bioavailable vitamin D and vitamin D-binding protein in early pregnancy with neonatal outcomes: A retrospective cohort study. Nutrients 2020, 12, 2495. doi:10.3390/nu12092495.
S23. Xia, J.; Song, Y.; Rawal, S.; Wu, J.; Hinkle, S.N.; Tsai, M.Y.; Zhang, C. Vitamin D status during pregnancy and the risk of gestational diabetes mellitus: A longitudinal study in a multiracial cohort. Diabetes Obes. Metab. 2019, 21, 1895–1905. doi:10.1111/dom.13748.
S24. George, J.A.; Norris, S.A.; Snyman, T.; Khambule, L.; Toman, M.; Crowther, N.J. Longitudinal changes in vitamin D and its metabolites in pregnant South Africans. J. Steroid Biochem. Mol. Biol. 2021, 212, 105949. doi:10.1016/j.jsbmb.2021.105949.
S25. Le, J.; Lv, Z.H.; Peng, R.; Li, Y.; Wang, S.T. Evaluation of vitamin D status and the analysis of risk factors of vitamin D deficiency in twin pregnancies. Lab. Med. 2023, 54, 534–542. doi:10.1093/labmed/lmad005.
S26. McWhorter, C.A.; Mead, M.J.; Rodgers, M.D.; Ebeling, M.D.; Shary, J.R.; Gregoski, M.J.; Newton, D.A.; Baatz, J.E.; Hollis, B.W.; Hewison, M.; et al. Predicting comorbidities of pregnancy: A comparison between total and free 25(OH)D and their associations with parathyroid hormone. J. Steroid Biochem. Mol. Biol. 2023, 235, 106420. doi:10.1016/j.jsbmb.2023.106420.
S27. Stunes, A.K.; Mosti, M.P.; Gustafsson, M.K.; Børsting, T.; Thorsby, P.M.; Stafne, S.N.; Syversen, U. Maternal vitamin A and D status in the second and third trimesters of pregnancy and bone mineral content in offspring at nine years of age. Front. Endocrinol. 2024, 15, 1417656. doi:10.3389/fendo.2024.1417656.
S28. Fakhoury, H.M.A.; Arabi, T.Z.; Tamim, H.; Chun, R.F.; Grant, W.B.; Hewison, M.; AlAnouti, F.; Pilz, S.; Annweiler, C.; Tzimagiorgis, G.; et al. Associations of free, bioavailable and total 25-hydroxyvitamin D with neonatal birth anthropometry and calcium homeostasis in mother–child pairs in a sunny Mediterranean region. Br. J. Nutr. 2024, 131, 801–808. doi:10.1017/S000711452300243X.
S29. Mercer, S.; Chen, X.; Tiwari, B.B.; Anderson, A.; Boulet, S.L.; Rajbhandari, J.; Gallo, S. Changes in vitamin D biomarkers across pregnancy and by maternal BMI: A secondary analysis of data and biospecimens from the National Children’s Study. J. Steroid Biochem. Mol. Biol. 2025, 252, 106791. doi:10.1016/j.jsbmb.2025.106791.
S30. Tian, Z.; Xu, X.; Gong, X.; Zeng, S.; Fu, S.; Chen, Y.; Yang, Y.; Sun, G.; Tian, M.; Li, J. Comparison study of the correlation between free and total 25(OH)D in maternal and umbilical blood and early-life physical development parameters. J. Pediatr. Endocrinol. Metab. 2025, 38, 1068–1077. doi:10.1515/jpem-2025-0001.
